# DNMT2‐m5C‐ACLY Axis Promotes Lenvatinib Resistance in Hepatocellular Carcinoma Through Histone Acetylation‐Mediated Notch Pathway

**DOI:** 10.1002/advs.202515931

**Published:** 2025-12-19

**Authors:** Shiguang Yang, Jie Li, Shengwei Mao, Xuhui Zhao, Douwaner Liu, Jiafeng Chen, Jialu Fu, Yichao Bu, Xiaoling Wu, Shaoqing Liu, Qifeng Yu, Qiongzhu Dong, Zheng Tang, Yuan Fang, Yinghong Shi

**Affiliations:** ^1^ Department of Hepatobiliary Surgery and Liver Transplantation Key Laboratory of Carcinogenesis and Cancer Invasion Liver Cancer Institute Zhongshan Hospital Fudan University Shanghai China; ^2^ Department of Hepatobiliary and Pancreatic Surgery Key Laboratory of Whole‐Period Monitoring and Precise Intervention of Digestive Cancer Minhang Hospital Fudan University Shanghai China; ^3^ National Clinical Research Center for Interventional Medicine Liver Cancer Institute Zhongshan Hospital Fudan University Shanghai China; ^4^ Department of Radiation Oncology Huadong Hospital Fudan University Shanghai China; ^5^ Department of Breast Surgery The First Affiliated Hospital Zhengzhou University Zhengzhou China; ^6^ Department of Hepatobiliary Surgery Zhoushan Hospital Zhoushan China

**Keywords:** hepatocellular carcinoma, histone acetylation, lenvatinib resistance, m5C modification

## Abstract

Lenvatinib serves as a first‐line therapy for advanced hepatocellular carcinoma (HCC), but its clinical efficacy is severely limited by acquired drug resistance. Consequently, the identification of therapeutic targets to reverse lenvatinib resistance may offer novel strategies to achieve enhanced and durable treatment responses. In this study, a global elevation in the levels of m5C modification is observed in lenvatinib‐resistant cells compared with their parental counterparts. DNA methyltransferase 2(DNMT2), functioning as an RNA methyltransferase, is markedly upregulated in lenvatinib‐resistant specimens and correlated with poor patient survival outcomes. Both in vitro and in vivo experiments indicated that DNMT2 downregulation effectively overcame lenvatinib resistance by reducing HCC cell proliferation and promoting apoptosis, thereby restoring drug sensitivity. Mechanistically, DNMT2 functioned together with Y‐box binding protein 1 (YBX1) to stabilize downstream adenosine triphosphate citrate lyase (ACLY) mRNA through m5C modification. This process activated the Notch signaling pathway by increasing intracellular acetyl‐CoA levels and promoting histone acetylation, driving the progression of lenvatinib resistance. Critically, pharmacological inhibition of ACLY combined with lenvatinib treatment enhanced the therapeutic efficacy against HCC and could reduce the tumor burden in multiple preclinical models. Collectively, the findings indicate the importance of the DNMT2‐ACLY‐NOTCH signaling axis in lenvatinib resistance and propose novel combinatorial therapies to improve HCC treatment outcomes.

## Graphical abstract

Lenvatinib resistance poses a major challenge in advanced hepatocellular carcinoma (HCC). This study reveals that DNMT2 upregulation is a key driver, which stabilizes ACLY mRNA via m5C modification and activates the Notch signaling pathway. Crucially, combining ACLY inhibitors with lenvatinib overcomes resistance and suppresses tumors, offering a promising new combination therapy for HCC.

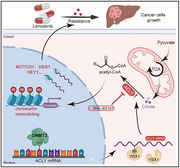

**Abbreviations**
ACLY: Adenosine triphosphate citrate lyase
DNMT2: DNA methyltransferase 2
HCC: Hepatocellular carcinoma
HES1: Hes family bHLH transcription factor 1
HEY1: Hes‐related family bHLH transcription factor with YRPW motif 1
TRDMT1: tRNA aspartic acid methyltransferase 1
YBX1: Y‐box binding protein 1

## 1 Introduction

Hepatocellular carcinoma (HCC) represents one of the most prevalent malignancies worldwide, ranking among the leading solid malignancies in terms of both incidence and mortality [1]. Clinically, approximately 70% of patients are diagnosed at intermediate or advanced stages, thus losing the opportunity for curative intervention. Lenvatinib, a first‐line standard therapy for advanced HCC, exhibits relatively favorable objective response and disease control rates. However, its clinical application faces the severe challenge of drug resistance, representing a critical bottleneck limiting treatment efficacy [2, 3]. Therefore, elucidating the molecular mechanisms underlying lenvatinib resistance and identifying novel therapeutic targets are critical for improving the prognosis of HCC patients.

In recent years, the relationship between RNA epigenetic modifications and cancer has attracted increasing attention. These modifications (including m6A, m5C, m7G, and ac4C) influence diverse processes such as splicing, nuclear localization, stability, and translation, thereby regulating vital cellular activities [4, 5, 6]. Specifically, m5C modification refers to the addition of an active methyl group onto cytidine residues. This modification occurs widely in messenger RNA (mRNA), ribosomal RNA (rRNA), and transfer RNA (tRNA), and is dynamically regulated by methyltransferases (writers), demethylases (erasers), and reader proteins [7, 8]. Many studies have documented associations between m5C modification and therapeutic resistance in various cancers, including gastric and ovarian cancer [9, 10, 11]. Nevertheless, there has been no specific investigation of the relationship between m5C modifications and lenvatinib resistance in HCC.

Acetyl‐coenzyme A (Ac‐CoA) occupies a central position at the intersection of metabolism and chromatin remodeling. Synthesized from precursors such as citrate or acetate, it forms a pivotal nexus integrating the metabolism of the three major nutrients: carbohydrates, lipids, and proteins [12]. Additionally, Ac‐CoA functions as an acetyl donor within the nucleus, participating in histone acetylation and regulating gene expression [13, 14]. In cancer cells, adenosine triphosphate citrate lyase (ACLY) is responsible for converting mitochondrial‐derived citrate into Ac‐CoA, and can promote aberrant proliferation through complex regulatory networks [15, 16]. Importantly, pharmacological ACLY inhibitors have generated increasing interest as promising anti‐cancer agents [17, 18, 19].

The present study established lenvatinib‐resistant HCC cell lines and observed a global elevation in m5C modification levels within these resistant cells. Moreover, DNA methyltransferase 2 (DNMT2) was identified as an upstream regulator of lenvatinib resistance, with high DNMT2 expression correlating with poorer survival prognosis in patients. Mechanistically, DNMT2‐mediated m5C modification was found to regulate the stability and expression of ACLY mRNA in a YBX1‐dependent manner, thereby increasing the levels of key histone acetylation modifications in components of the downstream Notch signaling pathway. Notably, pharmacological inhibition of ACLY combined with lenvatinib treatment enhanced the therapeutic efficacy against DNMT2‐high resistant HCC cells in preclinical models.

## 2 Materials and Methods

### 2.1 Clinical Patient Samples

This research included a total of 258 HCC patients from three cohorts at Zhongshan Hospital, Fudan University. Cohort 1 comprised 55 HCC patients who received lenvatinib treatment before the operation. Magnetic resonance imaging (MRI) examinations were performed every two months to evaluate treatment response. According to the modified RECIST criteria [20], complete response (CR) and partial response (PR) were considered indicative of lenvatinib sensitivity, whereas stable disease (SD) and progressive disease (PD) were classified as lenvatinib resistance.

Cohort 2 included samples from 173 patients who underwent curative hepatic resection between January 2012 and January 2013. Meanwhile, Cohort 3 consisted of tissue specimens from 30 HCC patients who were diagnosed and treated between January 2021 and May 2021, with all specimens stored at −80 °C. These patients did not receive neoadjuvant therapy prior to surgery.

### 2.2 Cell Culture

The human HCC cell lines Huh‐7 (CVCL_0336), PLC/PRF/5(CVCL_0485), and HEK293T(CVCL_0063) cells were obtained from the Shanghai Institute of Life Sciences, Chinese Academy of Sciences. All cell lines were confirmed to be free of mycoplasma contamination and were authenticated by short tandem repeat (STR) profiling.

All cell lines were cultured in medium supplemented with 10% fetal bovine serum and 1% penicillin‐streptomycin. Lenvatinib‐resistant cell lines were established using a gradually increasing concentration cultivation method. Huh7 and PLC/PRF/5 cells were initially cultured with 3 µmol/L and 20 µmol/L of lenvatinib, respectively. Following each passage and reseeding, the lenvatinib concentration was incrementally increased by 0.5‐1 µmol/L until robust proliferation was achieved at 30 µmol/L for Huh7 cells and 50 µmol/L for PLC/PRF/5 cells. These two cell lines were designated Huh7‐LR and PLC/PRF/5‐LR.

### 2.3 Animal Experiments

This research utilized 6‐week‐old BALB/c nude mice, C57BL/6 mice, and NOD‐SCID mice, which were purchased from Jiesijie and Charles River Laboratory Animal Company (Shanghai, China). For the orthotopic liver tumor model, nude mice were randomly divided into groups and anesthetized with tribromoethanol. Following midline laparotomy, 15 µL of Huh7‐LR cell suspension (approximately 5 × 10^6^ cells) was injected into each mouse's liver. After approximately two weeks, mouse livers were harvested, photographed, weighed, and volumetrically assessed. The tissues were subsequently fixed in 4% paraformaldehyde for immunohistochemical analysis.

For the spontaneous liver tumor model, the mice were given hydrodynamic tail vein injections (HTVIs) of 2 mL saline with the designated suspension (20µg pT3‐EF1α‐MYC‐IRES‐luciferase,20µg pT3‐EF1a‐NRas‐GV12, 2µg pCMV‐SB13). Animals were euthanized 5‐6 weeks post‐injection for tissue harvesting and analysis.

For the HCC patient‐derived xenograft (PDX) model, fresh tumor samples were preserved in pre‐cooled culture medium. The tissue was then cut into small fragments of 2–3 mm^3^ and implanted into the flanks of NOD/SCID mice. When the tumors had grown to a size of 1000–1500 mm^3^, they were re‐implanted into BALB/c nude mice.

For subsequent drug intervention experiments, approximately one week after the establishment of the tumor models, all mice were randomly divided into four groups: the control, BMS‐303141 (10 mg/kg, intraperitoneal injection once daily), lenvatinib (10 mg/kg, oral gavage once daily), and the combination therapy group. When the tumors in the PDX model mice reached a size of 50 mm^3^, the same treatment regimen was administered to these animals.

### 2.4 Statistical Analysis

Each independent experiment was repeated at least three times, and the results were expressed as mean±standard deviation(SD). Intergroup differences were analyzed using *t*‐test or two‐way ANOVA, and patient overall survival (OS) and recurrence‐free survival (RFS) were evaluated using the Kaplan‐Meier method. Statistical significance was defined as *P* <0.05.

The supplementary materials provide a detailed description of the other methods used in the study.

## 3 Results

### 3.1 m5C Hypermethylation and DNMT2 Overexpression are Associated With Lenvatinib Resistance in HCC

**FIGURE 1:**
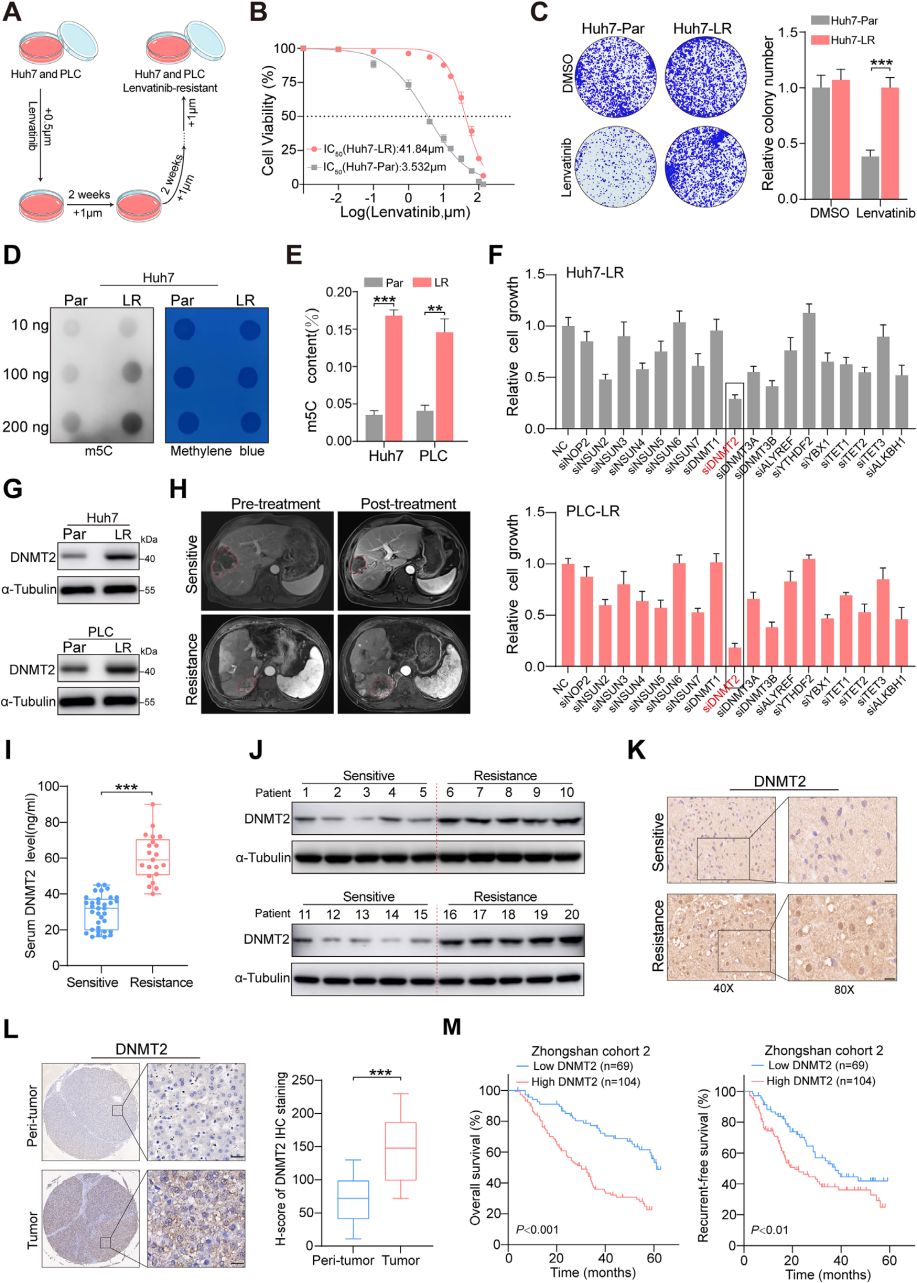
m5C hypermethylation and DNMT2 overexpression are associated with lenvatinib resistance in HCC. (A) Schematic workflow for generating drug‐resistant HCC cells. (B) Half‐maximal inhibitory concentration (IC_50_) curves of lenvatinib in parental Huh7 vs. lenvatinib‐resistant Huh7 cells. (C) Colony formation in parental and lenvatinib‐resistant Huh7 cells after lenvatinib treatment for 14 days (20 µm). (D‐E) Dot blot and ELISA quantification of global m5C modification levels in parental vs. lenvatinib‐resistant cells. (F) Effect of siRNA‐mediated knockdown of 18 m5C regulatory genes on the proliferation of resistant cells. (G) DNMT2 expression profiles in HCC cell lines. (H) Representative MRI images of patients sensitive and resistant to lenvatinib treatment. (I‐K) DNMT2 expression levels in patient cohorts receiving lenvatinib therapy(scale bar, 50 µm). (L) IHC staining and scoring of DNMT2 in HCC tissues vs. paratumor tissues (scale bar, 50 µm). (M) OS and RFS stratified by DNMT2 expression. ^*^ *p* <0.05; *p* <0.01; ^***^ *p* <0.001.

To elucidate the molecular mechanisms underlying resistance to lenvatinib in HCC, we first established two lenvatinib‐resistant cell lines (Huh7‐LR and PLC/PRF/5‐LR) by gradually increasing the drug dosage (Figure 1A). Compared to the parental cells, these resistant cells exhibited a higher IC_50_ value when exposed to lenvatinib treatment, accompanied by markedly enhanced cell viability (Figure 1B,C; Figure S1A,B). The role of m5C modification in the development and progression of various cancers is well‐documented [21, 22, 23]. Subsequently, both dot blot assays and ELISAs confirmed significantly elevated m5C methylation levels in lenvatinib‐resistant cell lines (Figure 1D,E; Figure S1C). This finding led to further investigation of the potential role of specific m5C regulators in HCC drug resistance. Small interfering RNAs (siRNAs) targeting 18 epigenetic regulatory factors associated with m5C modification were designed. Notably, we observed that downregulation of DNMT2 remarkably inhibited proliferation in resistant cells, leading to its selection as the focus of subsequent investigations (Figure 1F).

Subsequently, we confirmed that DNMT2 was highly expressed in the lenvatinib‐resistant cells (Figure 1G). To verify whether similar results existed in HCC patients, the expression levels of DNMT2 were assessed in a previously collected cohort (Cohort 1), which included 34 lenvatinib‐sensitive and 21 resistant patients (Figure 1H; Table S1). As expected, both serum and tissue samples from patients indicated that, compared to sensitive patients, those with resistance exhibited notably increased expression of DNMT2(Figure 1I–K; Figure S1D).

Furthermore, RNA sequencing data from the TCGA database indicated marked overexpression of DNMT2 in HCC, and patients with high DNMT2 expression showed significantly poorer OS than those with low DNMT2 expression (Figure S1E,F). Next, we validated the expression of DNMT2 using tissue microarrays comprising samples from 173 HCC patients (Cohort 2). Consistent with previous results, DNMT2 levels were enormously higher in tumor tissues compared to their paired adjacent non‐tumor tissues (Figure 1L). Moreover, elevated DNMT2 expression was associated with microvascular invasion, but showed no correlation with other clinical parameters such as patient age, gender, or tumor differentiation (Table S2). Similarly, patients with high DNMT2 expression exhibited significantly shorter OS and RFS than those with lower levels, and multivariable Cox regression confirmed that elevated DNMT2 expression was an independent predictor of poorer survival outcomes (Figure 1M; Figure S1G). Taken together, DNMT2‐mediated m5C modification may be associated with lenvatinib resistance and poor prognosis in HCC patients.

### 3.2 DNMT2 Modulates the Therapeutic Sensitivity of HCC Cells to Lenvatinib

**FIGURE 2:**
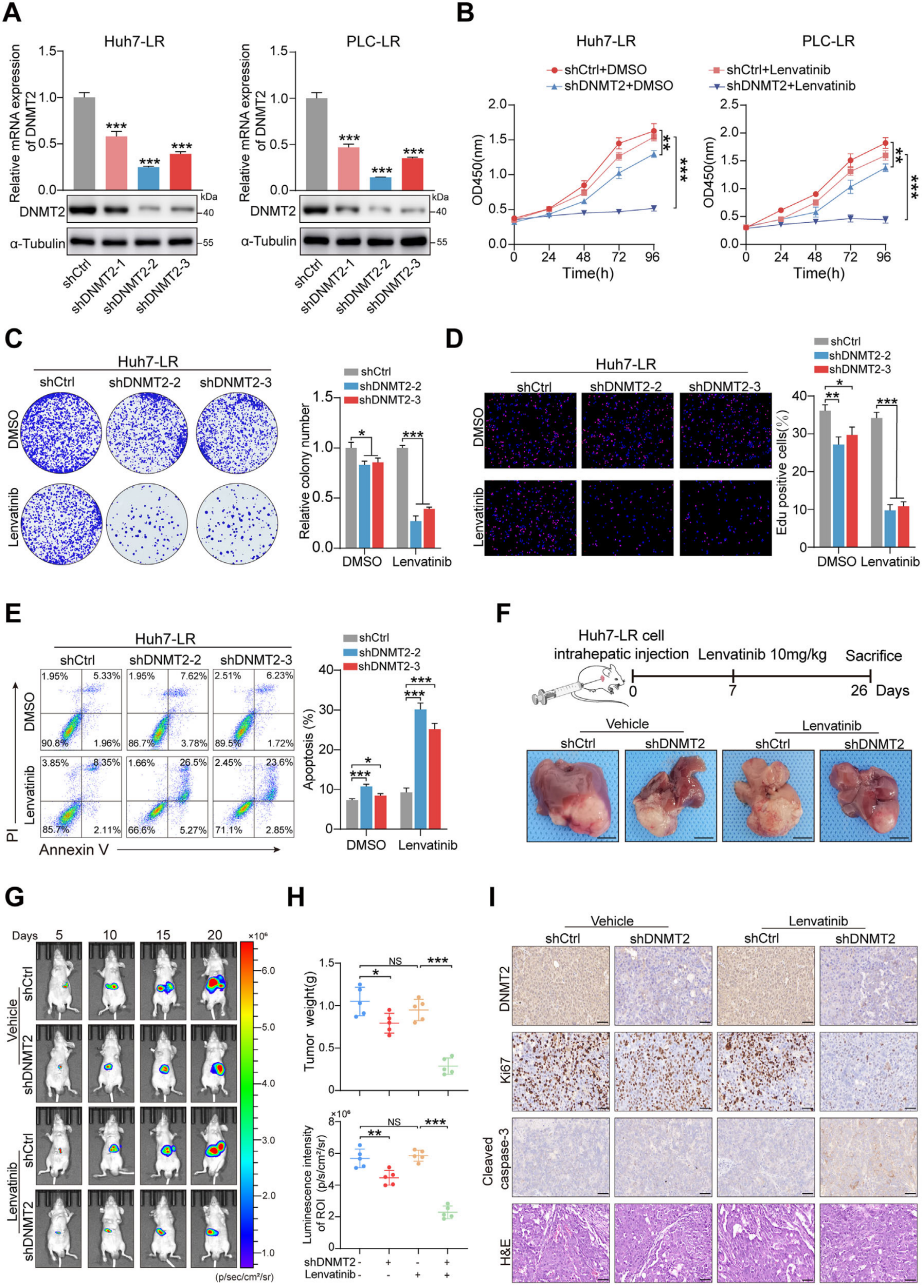
DNMT2 modulates the therapeutic sensitivity of HCC cells to lenvatinib. (A) Verification of transfection efficiency following DNMT2 knockdown in lenvatinib‐resistant cells. (B) Growth curves of resistant cells after DNMT2 knockdown. (C,D) Colony formation and EdU assays assessing changes in proliferation of Huh7‐LR cells after DNMT2 knockdown. (E) Flow cytometry analysis of Annexin V and PI staining in Huh7‐LR cells. (F) Representative images of liver tumors in the different groups (scale bar, 30 mm). (G) Longitudinal in vivo bioluminescence imaging at the indicated time points. (H) Tumor weight and bioluminescence imaging scores across the four groups. (I) Representative immunohistochemical images showing DNMT2, Ki67, and cleaved caspase‐3 expression in tumors from the four groups (scale bar, 50 µm). ^*^ *p* <0.05; ^**^ *p* <0.01; ^***^ *p* <0.001.

To further assess whether DNMT2 functions as a promoter of lenvatinib resistance in HCC, we employed a lentiviral system to establish two DNMT2‐knockdown‐resistant HCC cell lines (Figure 2A). Following DNMT2 knockdown, the IC_50_ values of Huh7‐LR and PLC‐LR cells were significantly reduced (Figure S1A,B). Additionally, treatment with lenvatinib alone did not markedly affect cell proliferation in Huh7‐LR and PLC‐LR cells, while silencing of DNMT2 sensitized resistant cells to lenvatinib, corresponding to impaired proliferation in growth curve analyses (Figure 2B). Likewise, colony formation and EdU assays further confirmed that DNMT2 knockdown reversed resistance to lenvatinib‐induced cell death, resulting in impaired proliferative capacity (Figure 2C,D; Figure S2C,D). Moreover, the combination of DNMT2 knockdown and lenvatinib treatment led to a rapid increase in the apoptosis rates of Huh7‐LR and PLC‐LR cells (Figure 2E; Figure S2E).

For subsequent functional experiments, we first overexpressed wild‐type DNMT2 or its catalytically inactive mutant (C79A) [24] in the parental Huh7 and PLC cells (Figure S3A). Overexpression of wild‐type DNMT2 markedly increased the IC_50_ value of lenvatinib in parental HCC cells (Figure S3B), enhanced cell proliferation capacity, and reduced the proportion of apoptotic cells (Figure S3C–H). However, the catalytically inactive DNMT2 had minimal effect on mediating the lenvatinib‐resistant phenotype (Figure S3C–H).

Next, we established an orthotopic liver tumor model to evaluate the in vivo therapeutic effects of targeting DNMT2 on lenvatinib resistance in HCC. Strikingly, inhibition of DNMT2 markedly potentiated the efficacy of lenvatinib in mice bearing Huh7‐LR cells, as shown by reduced intrahepatic tumor volumes and decreased tumor weights (Figure 2F–H; Figure S2F). Finally, immunohistochemical analysis indicated that the DNMT2‐knockdown group treated with lenvatinib exhibited lower expression of the proliferation marker Ki‐67 and increased expression of the apoptosis marker cleaved caspase‐3 (Figure 2I). These functional assays confirmed that DNMT2 plays a critical role in mediating lenvatinib resistance in HCC.

### 3.3 DNMT2 Enhances the Stability of ACLY mRNA Through m5C‐YBX1‐Dependent Methylation Modification

**FIGURE 3:**
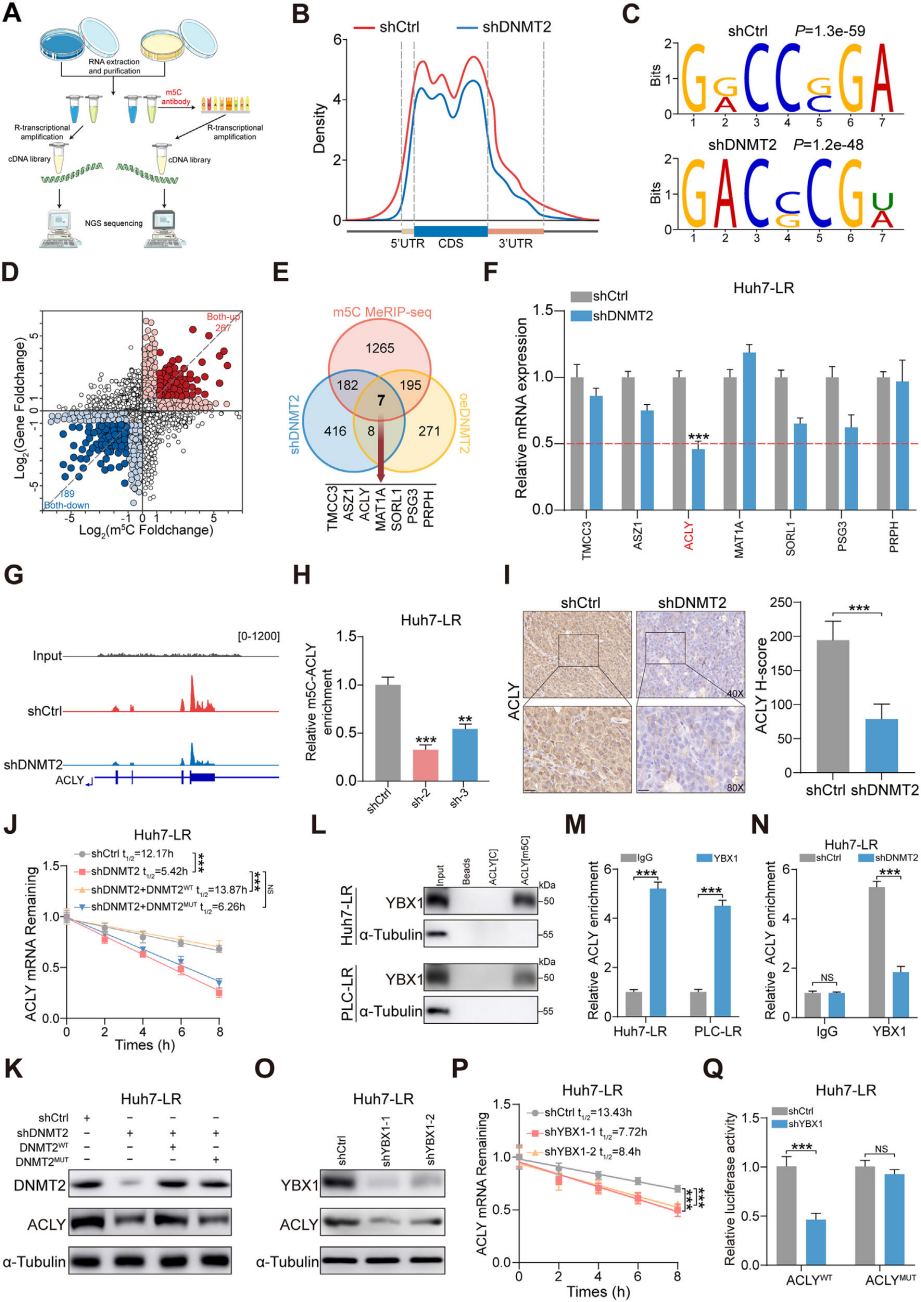
DNMT2 enhances the stability of ACLY mRNA through m5C‐YBX1‐dependent methylation modification. (A) Schematic overview of the MeRIP‐seq and RNA‐seq processes in HCC cells. (B) RNA‐m5C modification levels in control vs. DNMT2‐knockdown groups. (C) Consensus sequence of m5C modifications in Huh7‐LR cells. (D) Distribution maps of genes from MeRIP‐seq and RNA‐seq analyses. (E) Integrated analysis of MeRIP‐seq and RNA‐seq data to identify overlapping genes. (F) Expression changes in seven candidate target genes in Huh7‐LR cells after shDNMT2 treatment. (G) IGV analysis showing changes in ACLY m5C modification levels following DNMT2 knockdown. (H) MeRIP‐qPCR assessment of ACLY m5C modification abundance in DNMT2‐knockdown cells. (I) Immunohistochemistry analysis of the effect of DNMT2 knockdown on ACLY expression (scale bar, 50 µm). (J) Analysis of ACLY mRNA half‐life in Huh7‐LR cells treated with the different vectors. (K) WB analysis of ACLY protein expression in Huh7‐LR cells treated with the different vectors. (L,M) WB and RIP‐qPCR analyses demonstrating the potential interaction between YBX1 and m5C‐modified ACLY RNA. (N) RIP‐qPCR comparing YBX1 binding to ACLY RNA in control vs. DNMT2‐knockdown Huh7‐LR cells. (O,P) Effects of YBX1 downregulation on ACLY protein expression and RNA stability in Huh7‐LR cells. (Q) Relative luciferase activity of ACLY^WT^ and ACLY^MUT^ reporter vectors in control or YBX1 knockdown Huh‐LR cells. ^*^ *p* <0.05; ^**^ *p* <0.01; ^***^ *p* <0.001.

Considering that DNMT2 is a key m5C methyltransferase, we investigated how DNMT2 participates in the development of lenvatinib resistance in HCC through m5C‐dependent mechanism. Transcriptome sequencing and methylated RNA immunoprecipitation sequencing(MeRIP‐seq) were performed on Huh7‐LR cells with DNMT2 knockdown, overexpression, or corresponding control treatments (Figure 3A). As expected, MeRIP‐seq analysis revealed a trend toward decreased methylation levels across the entire genome in the DNMT2‐knockdown group (Figure 3B; Figure S4A). Additionally, the m5C modifications mediated by DNMT2 were primarily observed within “DCCC (D = U, A or G)” consensus motif (Figure 3C). To identify potential downstream target genes regulated by DNMT2, we conducted integrated analysis of the sequencing data and found that seven genes exhibited expression trends consistent with changes in DNMT2 (Figure 3D,E). Among these seven candidate genes, ACLY showed the greatest downregulation in Huh7‐LR and PLC‐LR cells following shDNMT2 treatment, indicating that it is a key target in the resistance mechanism(Figure 3F; Figure S4B). Furthermore, analysis of the TCGA database revealed a notable positive correlation between DNMT2 expression and ACLY expression in HCC, and elevated ACLY expression levels indicated poor prognosis in patients (Figure S4C–E).

Besides, Integrative Genomics Viewer (IGV) analysis demonstrated that DNMT2 knockdown resulted in reduced m5C modifications of ACLY transcripts (Figure 3G). Similarly, MeRIP‐qPCR confirmed an enormous reduction in m5C abundance on ACLY mRNA in DNMT2‐knockdown lenvatinib‐resistant cell lines (Figure 3H; Figure S4F). Immunohistochemical analysis confirmed that DNMT2 knockdown significantly reduced ACLY protein levels in Huh7‐LR xenograft tumors (Figure 3I). Additionally, RT‐qPCR and WB analyses demonstrated that DNMT2 knockdown markedly decreased ACLY mRNA and protein levels in HCC cells, whereas DNMT2 overexpression produced the opposite effects (Figure S4G,H). Given the functional properties of m5C modification, it is plausible that DNMT2 maintains ACLY expression through enhancing mRNA stability. Indeed, we observed that shDNMT2 markedly reduced both ACLY mRNA half‐life and protein expression, and this reduction could be rescued by wild‐type DNMT2 but not the catalytically inactive mutant (Figure 3J,K; Figure S4I,J). Integrated analysis based on MeRIP‐seq and bioinformatics revealed that DNMT2‐mediated m5C modifications are predominantly located within the coding sequence (CDS) of ACLY mRNA. Accordingly, we constructed luciferase reporter plasmids containing either the wild‐type (ACLY‐WT) or m5C‐site mutated (C1644A, C1771A, C1975A, and C2151A). Luciferase assays demonstrated that DNMT2 significantly enhanced the luciferase activity of ACLY‐WT, whereas this effect was markedly attenuated in the mutant construct (Figure S4K).

It is noteworthy that m5C modification also requires specific reader proteins to recognize and stabilize downstream target RNAs, Y‐box binding protein 1(YBX1) has been identified as a cytoplasmic reader protein, capable of binding to approximately 90% of m5C‐RNAs [25]. In line with these findings, YBX1 protein was successfully detected in the biotin‐labeled m5C‐ACLY RNA group, and RIP‐qPCR further confirmed the interaction between YBX1 and ACLY mRNA (Figure 3L,M). In the TCGA‐LIHC database, YBX1 and ACLY expression levels were significantly and positively correlated, whereas YBX1 expression remained unaltered in DNMT2‐downregulated HCC cell lines (Figure S5A,B). More specifically, RIP‐qPCR demonstrated substantially reduced binding between YBX1 and ACLY after the downregulation of DNMT2 (Figure 3N; Figure S5C). Considering the ability of YBX1 to bind and stabilize target m5C‐modified RNAs, we hypothesized that the enhanced stability of ACLY RNA mediated by DNMT2 at least partially depends on YBX1. Additionally, the RNA and protein levels of ACLY were crucially reduced following YBX1 knockdown in Huh7‐LR and PLC‐LR cells, and the half‐life of ACLY mRNA was also markedly shortened.

(Figure 3O,P; Figure S5D–F). Subsequently, we observed a significant reduction in YBX1‐RNA binding affinity following DNMT2 knockdown, this impaired interaction was rescued by wild‐type DNMT2, but not by the catalytically inactive mutant (Figure S5G,H). Dual‐luciferase reporter assays further demonstrated that YBX1‐knockdown reduced luciferase activity in the ACLY‐WT group, while having no significant effect on luciferase activity in cells transfected with the mutant ACLY plasmid (Figure 3Q; Figure S5I). Collectively, these data suggest that DNMT2 modulates ACLY mRNA stability by regulating YBX1 binding competence in lenvatinib‐resistant cells.

### 3.4 The DNMT2/ACLY Regulatory Axis Influences Lenvatinib Resistance in HCC

**FIGURE 4:**
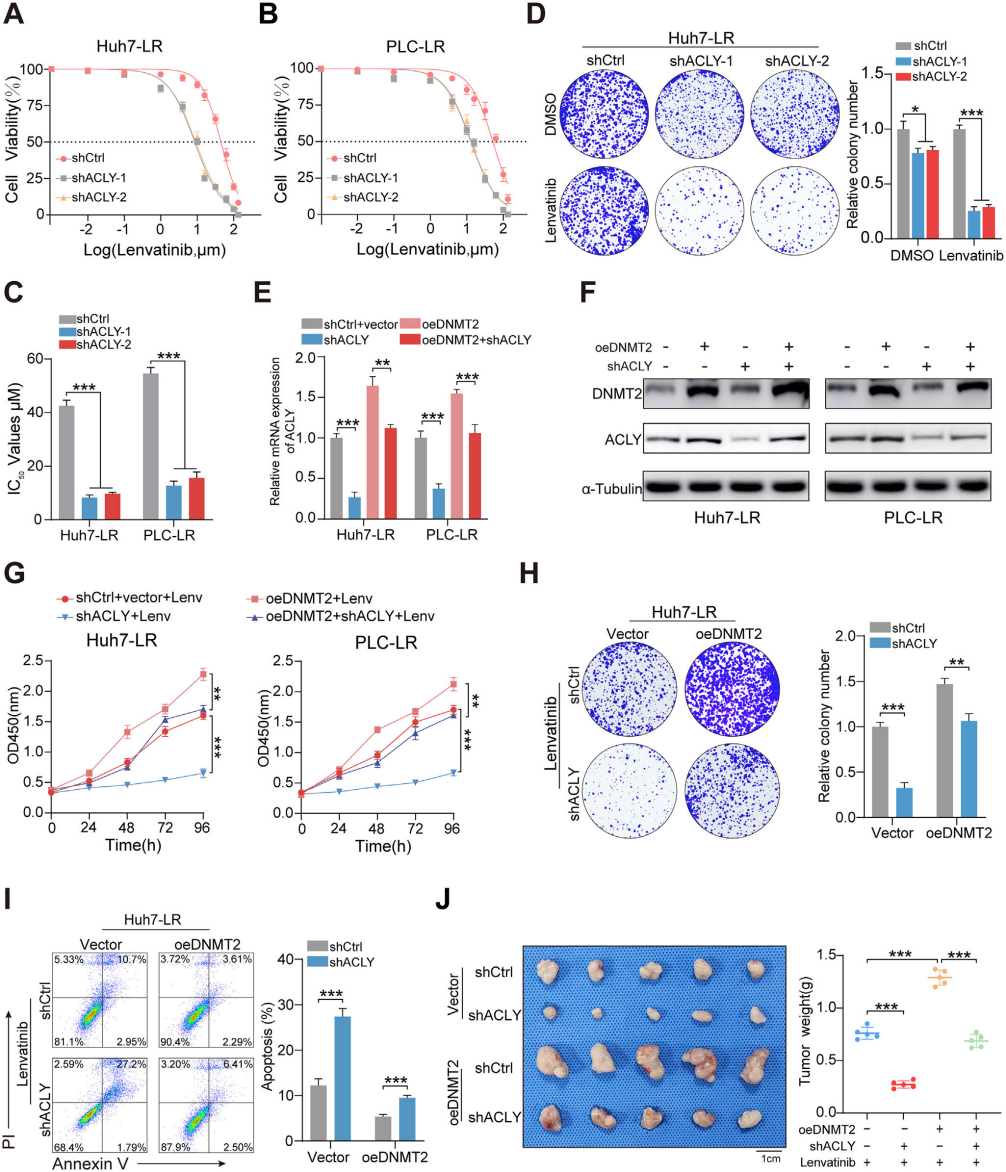
The DNMT2/ACLY regulatory axis influences lenvatinib resistance in hepatocellular carcinoma. (A–C) IC_50_ curves of parental and lenvatinib‐resistant cells following ACLY knockdown. (D) Colony formation assays assessing changes in the proliferation of Huh7‐LR cells after ACLY knockdown. (E,F) Effects of DNMT2 overexpression or ACLY knockdown on ACLY expression levels. (G,H) CCK‐8 and colony formation assays were conducted in Huh7‐LR cells with DNMT2 overexpression or ACLY knockdown. (I) Flow cytometry analysis of Annexin V and PI staining in Huh7‐LR cells with DNMT2 overexpression or ACLY knockdown. (J) Xenograft tumor growth was assessed following DNMT2 overexpression or ACLY knockdown. ^*^ *p* <0.05; ^**^ *p* <0.01; ^***^ *p* <0.001.

To investigate the role of ACLY in the efficacy of lenvatinib in HCC, we first silenced ACLY expression in Huh7‐LR and PLC‐LR cells, followed by a series of cellular functional assays (Figure S5A). Similarly, the downregulation of ACLY considerably reduced the IC_50_ values of resistant cells, while also attenuating the proliferation of Huh7‐LR and PLC‐LR cells following lenvatinib treatment(Figure 4A–D; Figure S6B). DNMT2 exerted a regulatory effect on ACLY expression, whereas ACLY did not influence DNMT2 expression levels, which experimentally confirmed their unidirectional regulatory relationship (Figure 4E,F). Next, rescue experiments were performed to further elucidate the contribution of the DNMT2/ACLY axis in lenvatinib resistance. Notably, knockdown of ACLY successfully reversed the increased proliferation capacity of Huh7‐LR and PLC‐LR cells mediated by DNMT2 overexpression (Figure 4G,H; Figure S6C). Furthermore, this knockdown also rescued the diminished rate of apoptosis induced by elevated DNMT2 expression (Figure 4I; Figure S6D). In vivo verification showed that tumors with stable DNMT2 overexpression exhibited increased growth, but this effect could be reversed upon ACLY knockdown, indicating that ACLY is a key downstream target of DNMT2 in HCC (Figure 4J; Figure S6E). Overall, these results highlight the novel role of the DNMT2/ACLY axis in mediating lenvatinib resistance, which may facilitate the development of targeted therapeutic strategies in the future.

### 3.5 The DNMT2/ACLY Axis Promotes Lenvatinib Resistance by Modulating Histone Acetylation

**FIGURE 5:**
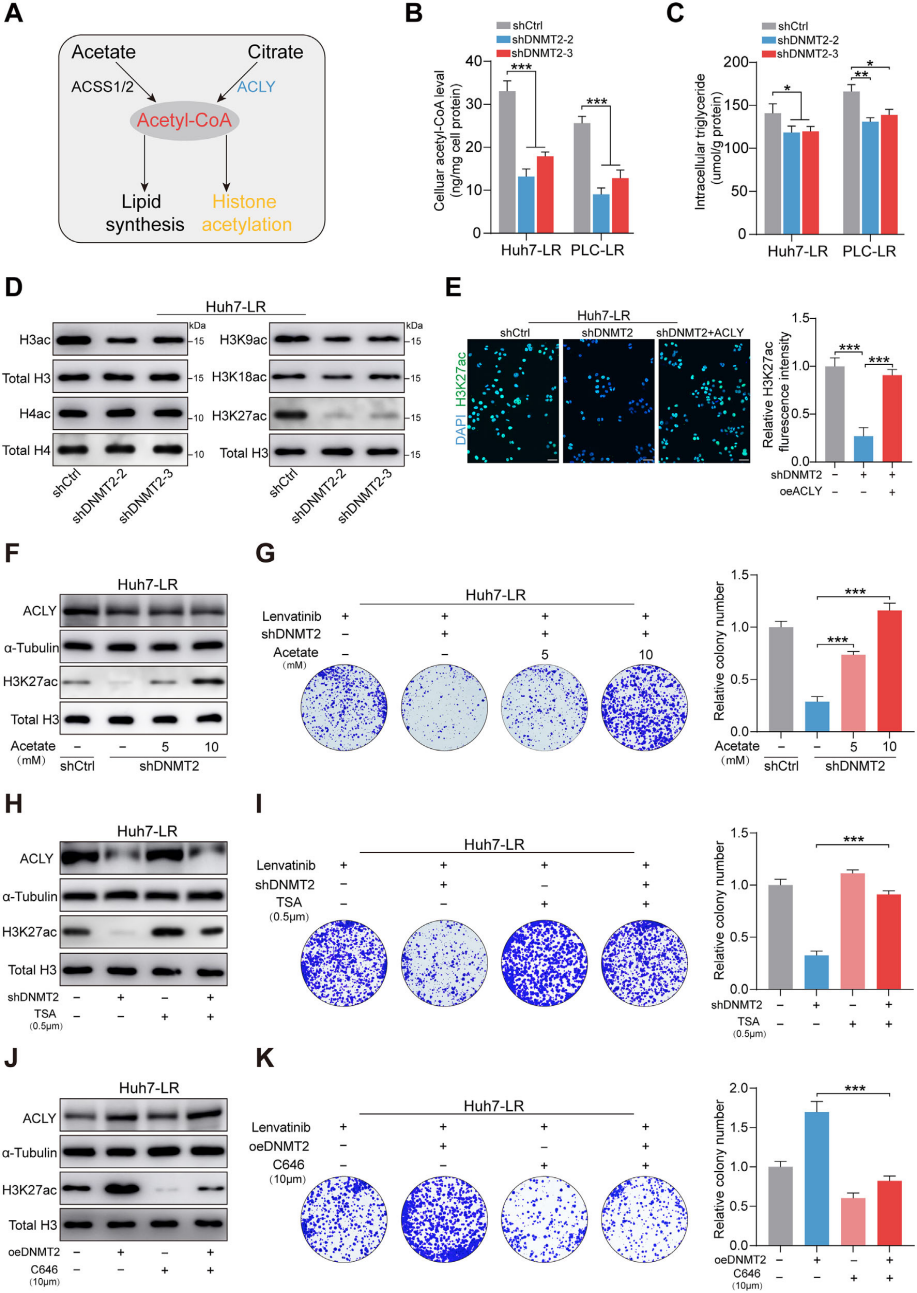
The DNMT2/ACLY axis promotes lenvatinib resistance by modulating histone acetylation. (A) Metabolic diagram of acetyl‐CoA in mammalian cells. (B,C) Effects of DNMT2 knockdown on acetyl‐CoA and triglyceride levels in drug‐resistant cells. (D) Levels of histone H3 and histone H4 acetylation in Huh7‐LR cells following DNMT2 knockdown. (E) Effects of DNMT2 knockdown or ACLY overexpression on H3K27ac staining (scale bar, 100 µm). (F,G) Impact of DNMT2 knockdown or exogenous acetate addition on H3K27 acetylation and cell viability in Huh7‐LR cells. (H,I) Effects of DNMT2 knockdown or TSA treatment on H3K27 acetylation and cell viability in Huh7‐LR cells. (J,K) Effects of DNMT2 overexpression or C646 treatment on H3K27 acetylation and cell viability in Huh7‐LR cells. ^*^ *p* <0.05; ^**^ *p* <0.01; ^***^ *p* <0.001.

As demonstrated in previous studies, ACLY is responsible for converting citrate into Ac‐CoA during cellular metabolism, participating in lipid synthesis and histone acetylation (Figure 5A). Initial observations revealed that downregulation of DNMT2 markedly reduced intracellular Ac‐CoA levels, a metabolic alteration that was subsequently rescued by ACLY overexpression (Figure 5B; Figure S7B). Thus, given that the levels of acetyl‐CoA synthetase short‐chain family member 2 (ACSS2) remained virtually unchanged, these findings substantiate the functional role of the DNMT2/ACLY axis in regulating Ac‐CoA provision (Figure S7A). Following DNMT2 knockdown, alterations in both cellular lipid content and global histone acetylation were observed. Specifically, intracellular levels of triglycerides and cholesterol exhibited only minor fluctuations (Figure 5C; Figure S7C,D). Furthermore, while DNMT2 knockdown had no significant effect on histone H4 acetylation, it markedly reduced acetylation of histone H3, with the most pronounced decrease occurring at the H3K27 site (Figure 5D; Figure S7E). Subsequent immunofluorescence analysis revealed that DNMT2 knockdown significantly reduced histone H3K27 acetylation (H3K27ac), and this reduction was effectively rescued by ACLY overexpression (Figure 5E; Figure S7F).

To thoroughly confirm the role of histone acetylation in lenvatinib resistance, we supplemented exogenous acetate at varying concentrations to elevate global histone acetylation levels following DNMT2 knockdown. Experimental results confirmed that DNMT2‐knockdown resistant cells exhibited progressively enhanced proliferation under continuous supplementation of exogenous acetate, accompanied by a concomitant reduction in apoptosis (Figure 5F,G; Figure S7G,H; Figure S8A). Similarly, it was observed that the histone deacetylase inhibitor trichostatin A (TSA) effectively inhibited the suppressive effect of shDNMT2 on both H3K27ac and cellular proliferation in Huh7‐LR and PLC‐LR cells (Figure 5H,I; Figure S8B,C). In contrast, C646, a histone acetyltransferase inhibitor, greatly attenuated the increase in H3K27ac induced by DNMT2 overexpression, thereby modulating proliferation of the resistant cells (Figure 5J,K; Figure S8D,E). Overall, these data indicate that the elevation of H3K27ac is essential for conferring lenvatinib resistance via the DNMT2/ACLY axis.

### 3.6 H3K27 Acetylation Epigenetic Remodeling Activates the Transcription of the Notch Signaling Pathway

**FIGURE 6:**
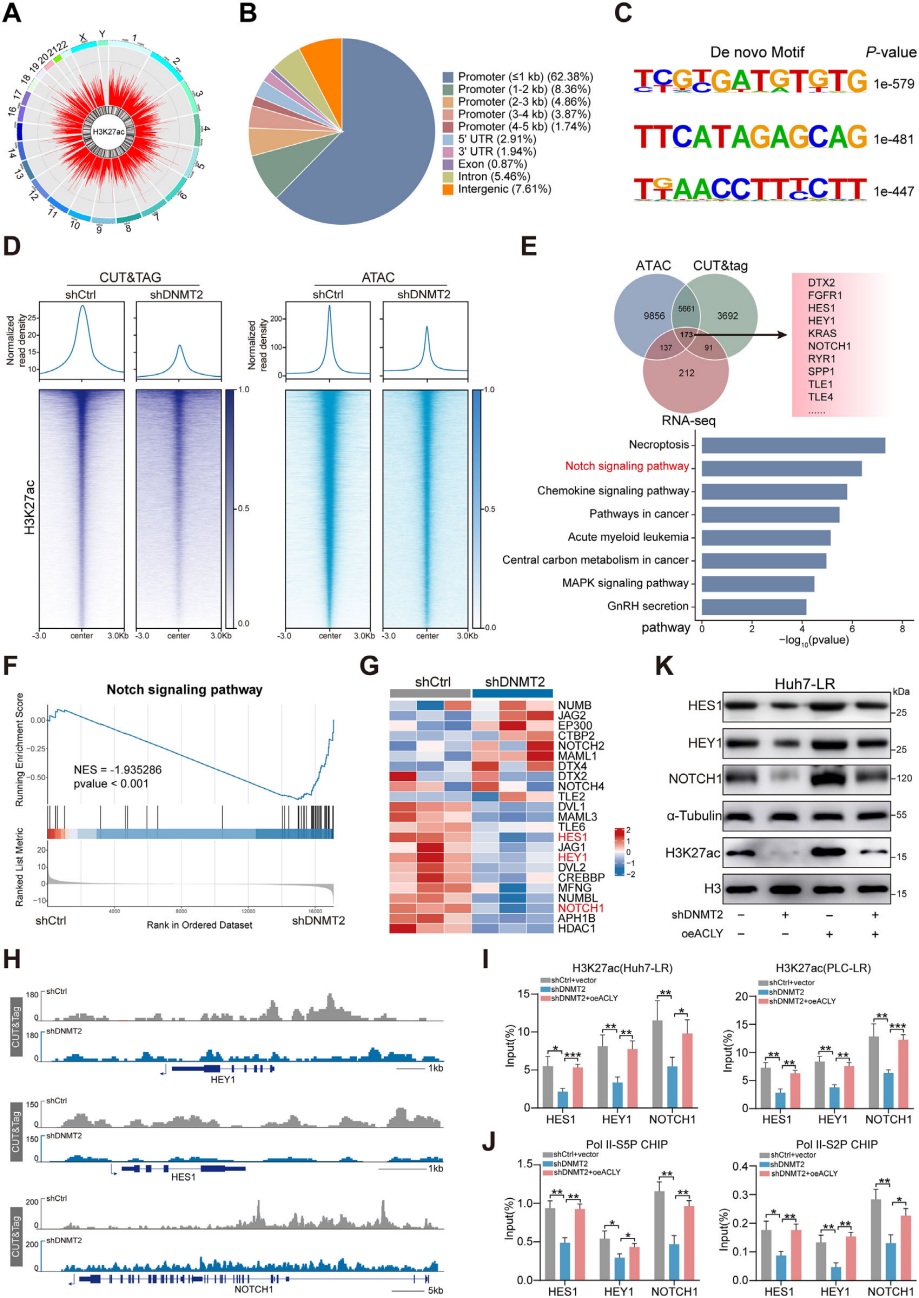
H3K27 acetylation epigenetic remodeling activates the transcription of the Notch signaling pathway. (A,B) Chromosomal distribution and genomic annotation of H3K27ac. (C) De novo motif analysis derived from H3K27ac CUT&Tag sequencing. (D) Profile plot and heatmap of CUT&Tag‐seq signals for H3K27ac and ATAC‐seq signals representing open chromatin in Huh7‐LR cells. (E) Venn diagram showing the overlapping genes among CUT&Tag‐seq, ATAC‐seq, and RNA‐seq datasets, with KEGG pathway analysis of the overlapping genes. (F) GSEA results indicating the association between DNMT2 expression and the Notch signaling pathway. (G) Heatmap depicting expression levels of Notch pathway‐related genes following DNMT2 knockdown. (H) WB analysis of NOTCH1, HES1, and HEY protein expression after DNMT2 knockdown or ACLY overexpression in Huh7‐LR cells. (I) Visualization of peaks from CUT&Tag‐seq demonstrating H3K27ac binding at promoters of Notch1, HES1, and HEY genes. (J) ChIP‐qPCR analysis of H3K27ac modifications at the promoters of indicated genes following DNMT2 knockdown or ACLY overexpression. (K) ChIP‐qPCR analysis of Pol II‐S5P and S2P occupancy at the promoter regions of the indicated genes after DNMT2 knockdown or ACLY overexpression. ^*^ *p* <0.05; ^**^ *p* <0.01; ^***^ *p* <0.001.

To systematically investigate the specific role of H3K27ac modification mediated by the DNMT2/ACLY axis in lenvatinib resistance in HCC, we performed H3K27ac CUT&Tag and ATAC assays to profile their distribution in shDNMT2‐treated Huh7‐LR cells. The CUT&Tag analysis revealed widespread distribution of H3K27ac marks across the genome, with approximately 80% concentrated within promoter regions (Figure 6A,B). Subsequently, de novo motif analysis identified the top three motifs closely associated with H3K27ac occupancy (Figure 6C). Further sequencing analysis showed that DNMT2 knockdown significantly reduced the levels of H3K27ac modification, as well as chromatin accessibility at 39,753 genome sites (Figure 6D). Strikingly, integrated RNA‐seq, CUT&Tag‐seq, and ATAC‐seq analysis identified 173 differentially expressed genes, predominantly enriched in the Notch signaling pathway (Figure 6E,F). Heatmap analysis demonstrated that when DNMT2 expression levels decreased, the expression of genes such as NOTCH1, HES1, and HEY1 in the Notch signaling pathway was markedly downregulated (Figure 6G). More importantly, CUT&Tag‐seq and ATAC‐seq analyses indicated that DNMT2 knockdown significantly reduced chromatin accessibility at NOTCH1, HES1, and HEY, as well as decreased H3K27ac enrichment in the promoters of these genes, providing an explanation for the observed downregulation of their expression levels (Figure 6H; Figure S9C). Consistent with the sequencing results, ChIP‐qPCR showed that inhibition of DNMT2 expression reduced H3K27ac levels at the transcription start sites of NOTCH1, HES1, and HEY1, and this effect was rescued by ACLY overexpression (Figure 6I). The C‐terminal domain (CTD) of RNA polymerase II (RNA Pol II) is a dynamic structure composed of highly repetitive heptapeptide sequences, and its core function involves the precise recruitment and coordination of transcription through reversible phosphorylation of key residues such as Ser2, Ser5, Ser7, and Thr4 [26, 27]. Furthermore, ChIP‐qPCR analysis revealed that downregulation of DNMT2 resulted in decreased levels of Ser5‐ and Ser2‐phosphorylated RNA Pol II CTD at NOTCH1, HES1, and HEY1, indicating impaired transcriptional initiation and elongation. Importantly, overexpression of ACLY successfully rescued these alterations induced by shDNMT2 (Figure 6J). Besides, RT‐qPCR and WB demonstrated that DNMT2 knockdown markedly decreased the expression of NOTCH1, HES1, and HEY1 in resistant cells, which was rescued by elevated ACLY expression (Figure 6K; Figure S9A,B). Colony formation assays demonstrated that inhibition of the Notch signaling pathway successfully reversed the enhanced proliferative capacity mediated by DNMT2 overexpression in HCC cells (Figure S9D,E). Conclusively, the DNMT2/ACLY axis influences the level of H3K27ac at transcriptional regulatory regions of Notch pathway genes, thereby contributing to the lenvatinib resistance process.

### 3.7 The ACLY Inhibitor Enhances the Therapeutic Efficacy of Lenvatinib in Multiple Preclinical HCC Models

**FIGURE 7:**
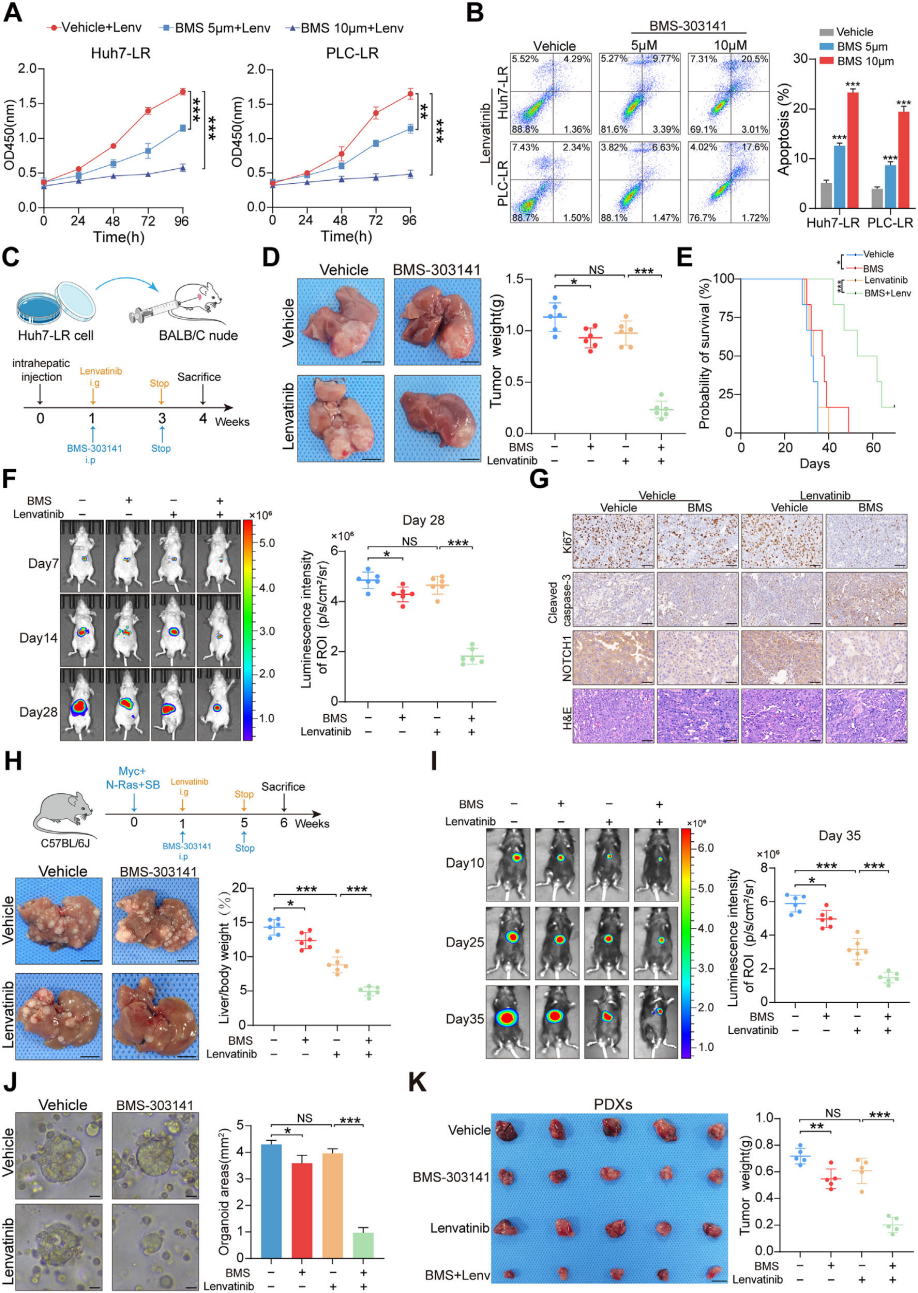
The ACLY inhibitor enhances the therapeutic efficacy of lenvatinib in multiple preclinical HCC models. (A,B) Effects of combined BMS‐303141 and lenvatinib treatment on proliferation and apoptosis of resistant cells. (C–F) Impact of combined BMS‐303141 and lenvatinib on tumor proliferation and survival rates in mouse orthotopic liver cancer models (n = 6, scale bar,0.5 cm). (G) Immunohistochemical analysis of Ki‐67, cleaved caspase‐3, and NOTCH1 in tissue specimens from the orthotopic liver cancer model (scale bar, 50 µm). (H,I) Effects of combined BMS‐303141 and lenvatinib on tumor proliferation in spontaneous liver cancer mouse models(n = 6). (J) Impact of combined BMS‐303141 and lenvatinib on proliferation in patient‐derived organoid models constructed from lenvatinib‐resistant specimens(scale bar, 500 µm). (K) Effects of combined BMS‐303141 and lenvatinib on tumor proliferation in lenvatinib‐resistant PDX models(scale bar, 1 cm). ^*^ *p* <0.05; ^**^ *p* <0.01; ^***^ *p* <0.001.

BMS‐303141, an inhibitor of ACLY, can effectively influence its biological activity. In vitro experiments demonstrated that the addition of BMS‐303141 markedly restored the growth‐inhibitory effect of lenvatinib on cells, increasing the proportion of apoptotic cells, and this effect was dose‐dependent on BMS‐303141 concentration(Figure 7A,B; Figure S10A). To investigate the impact of BMS‐303141 on lenvatinib resistance in vivo, we established orthotopic liver tumor models in nude mice using Huh7‐LR cells and administered drug treatments (Figure 7C). The results demonstrated that, compared to treatment with BMS‐303141 or lenvatinib alone, the combination therapy notably enhanced the anti‐tumor effect, resulting in dramatic reductions in tumor volume and weight, as well as prolonged survival of the mice (Figure 7D–F; Figure S10B). Immunohistochemical results also indicated that the combined use of BMS‐303141 and lenvatinib considerably affected tumor proliferation marker Ki‐67 and apoptosis marker cleaved caspase‐3, further confirming the sensitizing effect of BMS on lenvatinib (Figure 7G). Additionally, together with modulating ACLY expression, BMS effectively suppressed the expression of genes within the Notch signaling pathway, including NOTCH1, HES1, and HEY1 (Figure 7G; Figure S10C). Next, we constructed spontaneous HCC models by HTVIs of N‐Ras/c‐Myc luciferase plasmids. The combined treatment with BMS‐303141 and lenvatinib resulted in the most significant inhibition of tumor growth, and the survival times of the mice were also notably prolonged (Figure 7H,I; Figure S10D). Moreover, the combination therapy exhibited no detectable drug‐related toxicity in hepatic or renal function, supporting its favorable safety profile (Figure S10E).

Patient‐Derived Organoid(PDO) and PDX models provide comprehensive simulations of the human tumor environment, offering significant advantages for elucidating resistance mechanisms and accelerating drug development. The HCC‐PDO model revealed that, compared to monotherapy, the combination of BMS‐303141 and lenvatinib substantially suppressed the growth of resistant tumors (Figure 7J). Additionally, we established PDX models using resistant tumor tissues, showing that BMS‐303141 enhanced the therapeutic efficacy of lenvatinib and reduced tumor burden (Figure 7K; Figure S10F,G). In summary, the collective findings from these therapeutic models substantiate the crucial role of BMS‐303141 in enhancing the efficacy of lenvatinib in DNMT2‐high resistant HCC, thereby providing a mechanistic foundation for the development and application of effective therapeutic agents.

### 3.8 The Co‐Expression of DNMT2 and ACLY is Associated with Poor Prognosis in HCC Patients

**FIGURE 8:**
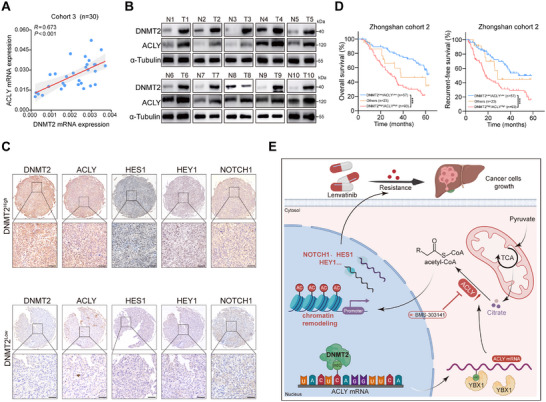
The co‐expression of DNMT2 and ACLY is associated with poor prognosis in HCC patients. (A,B) Correlation between RNA and protein expression levels of DNMT2 and ACLY. (C) Immunohistochemical detection of DNMT2, ACLY, HES1, HEY1, and NOTCH1 expression in tissue microarrays (scale bar, 50 µm). (D) Impact of DNMT2 and ACLY expression on patient OS and RFS. (E) Overall Schematic diagram of this article. ^*^ *p* <0.05; ^**^ *p* <0.01; ^***^ *p* <0.001.

Although the clinical correlation between DNMT2 and ACLY was preliminarily verified in data from public databases, we also collected clinical specimens from Zhongshan Hospital, Fudan University, to provide direct evidence. First, RT‐qPCR results of 30 HCC samples from Cohort 3 showed a significant positive correlation between the mRNA expression levels of DNMT2 and ACLY, and WB analyses of 10 paired tumor and normal tissue samples yielded similar results (Figure 8A,B). To further verify the correlation between the expression of DNMT2, ACLY, NOTCH1, HES1, and HEY1 in HCC tissues, immunohistochemical staining was performed on 173 HCC tissue microarrays from Cohort 2. These results revealed a coordinated expression pattern in which elevated co‐expression of DNMT2 and ACLY was correlated with upregulation of NOTCH1, HES1, and HEY1 (Figure 8C; Figure S10H). Moreover, patients with high expression of both DNMT2 and ACLY exhibited poorer OS and RFS, suggesting that the synergistic action of these molecules may be closely associated with adverse prognosis in HCC (Figure 8D).

## 4 Discussion

Although lenvatinib represents a cornerstone of systemic therapy for advanced HCC, most patients develop acquired resistance within 6‐10 months of treatment, substantially limiting its therapeutic efficacy [28]. In this study, we identified significant alterations in global m5C modification levels between lenvatinib‐resistant HCC cell lines and their parental counterparts. Using siRNA library screening, DNMT2 was identified as a critical epigenetic regulator of lenvatinib resistance. Knockdown of DNMT2 notably enhanced the cytotoxic effects of lenvatinib in resistant cells. Additionally, overexpression of wild‐type DNMT2, but not its catalytically inactive mutant, conferred resistance to lenvatinib. Mechanistically, DNMT2 was found to upregulate ACLY expression via YBX1‐dependent m5C modification, thereby driving elevated global histone acetylation. This epigenetic remodeling subsequently modulated the levels of key target components of the Notch signaling pathway. Furthermore, the small‐molecule ACLY inhibitor BMS‐303141 markedly overcame lenvatinib resistance and potentiated the anti‐tumor efficacy (Figure 8E). These findings elucidate the crosstalk between m5C modification, histone remodeling, and lenvatinib resistance in HCC, identifying novel therapeutic targets to overcome resistance.

DNMT2, also commonly referred to as tRNA aspartic acid methyltransferase 1 (TRDMT1), is a strongly conserved methyltransferase. Although its name derives from its sequence similarity to the DNA methyltransferase family, in‐depth studies have shown that DNMT2 does not directly act on DNA but instead specifically catalyzes the transfer of methyl groups to RNA molecules(tRNA and mRNA) [29, 30]. Furthermore, DNMT2 plays critical roles in regulating cellular senescence, oxidative stress, and tumor progression, positioning it as a viable target for anti‐cancer strategies [31, 32]. In addition to the increased sensitivity of DNMT2‐knockout osteosarcoma cells to radiotherapy, the loss of DNMT2 methyltransferase activity can lead to the degradation of certain mutant proteins and enhance the sensitivity of ovarian cancer to cisplatin [24, 33]. Consistent with previous research findings, this study observed that reduced expression of DNMT2 can effectively overcome lenvatinib resistance and enhance its therapeutic efficacy in HCC. Besides, clinical samples analysis indicated that DNMT2 may represent a valuable prognostic biomarker for HCC patients.

As a key molecule at the intersection of lipid metabolism and epigenetic modification, ACLY can drive vascular remodeling and tumor immune evasion by influencing histone acetylation [17, 19]. Critically, the present study found that ACLY was a key downstream target of DNMT2‐mediated m5C modification, with the DNMT2/ACLY axis promoting lenvatinib resistance through histone acetylation‐dependent mechanisms. Histone acetylation is a dynamic, reversible epigenetic modification that regulates chromatin structure and controls transcriptional initiation/elongation, gene silencing, and epigenetic cellular memory [34, 35, 36]. This study observed a global increase in histone acetylation levels, particularly H3K27ac, which prompted further identification of its downstream activation targets using high‐throughput sequencing. The Notch signaling pathway, known to regulate cell differentiation, organ development, and tissue homeostasis, has been strongly conserved throughout evolution. Its regulatory function in cancer metabolic reprogramming and the tumor microenvironment represents a key mechanism balancing tumorigenesis and tumor suppression [37, 38]. Additionally, the Notch pathway contributes to the maintenance of stem‐like characteristics in cancer cells and is associated with tumor cell targeting and chemoresistance [39, 40, 41]. Our findings demonstrated that DNMT2/ACLY upregulated H3K27ac levels at the promoter regions of core components of the Notch pathway (e.g., NOTCH1, HES1, and HEY1), enhancing their transcription through histone epigenetic remodeling.

Currently, numerous inhibitors targeting the Notch signaling pathway are under clinical investigation for malignant tumors [42, 43, 44]. Although this aspect has not been systematically explored in this study, preliminary in vitro experimental results suggest that combining Notch signaling inhibitors with lenvatinib may enhance therapeutic efficacy.

Based on the above, we observed that DNMT2 regulates the stability of ACLY mRNA through m5c modification, leading to an increase in the acetylation levels of key proteins in the Notch signaling pathway, subsequently inducing lenvatinib resistance in HCC. This study uncovers a novel mechanism underlying lenvatinib resistance in HCC and provides a compelling reference for the identification of potential targets to overcome such resistance.

## Author Contributions

S. Y., J. L., and S. M. contributed equally to this work. S.Y. contributed to writing the original draft, data curation, and formal analysis. J.L. was responsible for methodology development, software implementation, and project administration. S.M. carried out the investigation and contributed to software development, while X.Z. and X.W. performed formal analysis. D.L. contributed to the conceptualization of the study, and J.C. supported the methodology. J.F. was responsible for visualization, and Y.B. conducted validation. S.L. contributed to software development, and Q.Y. assisted with data curation. Q.D. secured funding for the project. Z.T. provided project administration and supervision. Y.F. contributed to the investigation and visualization. Y.S. was responsible for funding acquisition, supervision, and writing, review, and editing.

## Funding

This work was supported by the National Natural Science Foundation of China (No. 82273386, 82273387, 82403555, and 82573205), Clinical research projects of Zhongshan Hospital Affiliated to Fudan University (No. H2021‐021), and the Youth Fund of Zhongshan Hospital Affiliated to Fudan University (No. 2023ZSQN16 and 2023ZSQN47).

## Ethical Approval

The collection of clinical samples was approved by the Clinical Research Ethics Committee of Zhongshan Hospital, Fudan University (Approval No. B2021‐143R), and informed consent was obtained from all patients in accordance with the principles of the Declaration of Helsinki. All animal experiments were conducted in compliance with ethical guidelines and were approved by the Animal Experiment Ethics Committee of Zhongshan Hospital, Fudan University (Approval No.20220120‐068).

## Conflicts of Interest

The authors declare no conflicts of interest.

## Supporting information




**Supporting file**: advs73473‐sup‐0001‐SuppMat.docx

## Data Availability

Relevant data supporting the findings of this study can be reasonably requested from the corresponding author.
