# Supplementary material for: DNMT2‐m5C‐ACLY Axis Promotes Lenvatinib Resistance in Hepatocellular Carcinoma Through Histone Acetylation‐Mediated Notch Pathway
Source: Adv Sci (Weinh). 2025 Dec 19;13(13):e15931. doi: 10.1002/advs.202515931 (PMC12955885; doi:10.1002/advs.202515931)
Supplement: Supplementary file 1 — Supporting file: advs73473‐sup‐0001‐SuppMat.docx [file ADVS-13-e15931-s001.docx]

**Supplementary Materials and methods**

**Cell Counting Kit-8 assay(CCK-8)**

Cells in the logarithmic growth phase were seeded into 96-well plates at an appropriate density (2×10³ to 5×10³ cells per well), as determined by preliminary experiments. After cell attachment, different concentrations of the test drugs were added according to the experimental design. At designated time points (e.g., 0, 24, 48, 72 hours), 10 μL of CCK-8 solution was added to each well. Following incubation in the dark for 1–4 hours, the optical density (OD) values of each well were measured at 450 nm using a microplate reader,Cell viability (%) = [(ODtreated - ODblank) / (ODcontral - ODblank)]×100%.

**Colony Formation and 5-Ethynyl-2'-deoxyuridine Incorporation (EdU) Assay**

Depending on the cell type and proliferative capacity, an appropriate number of cells were seeded into 6-well plates and gently agitated to ensure even distribution. Cells were cultured under standard conditions (37°C, 5% CO₂) for approximately 7 to 14 days, with regular replacement of fresh culture medium. At the end of the culture period, the medium was discarded, and the cells were gently rinsed 1-2 times with PBS. Then, an appropriate amount of 4% paraformaldehyde was added to fix the cells for 15 minutes. After removing the fixative and washing with PBS, crystal violet staining solution (0.1%) was applied to stain the cells for 20 minutes. Finally, excess dye was rinsed off slowly with running water, and the plates were dried at room temperature.

The EdU assay kit was purchased from Beyotime Biotechnology Co., Ltd(Shanghai,China),and all experimental procedures were carried out step-by-step according to the instruction manual. Finally, images of randomly selected fields of view were captured and documented using a fluorescence microscope.

**m5C Dot Blot assay**

Total RNA was extracted from lenvatinib-resistant cells, and the nucleic acid concentration and purity were measured. A 1–2 μL aliquot of the diluted sample was spotted onto a positively charged nylon membrane, followed by UV crosslinking for 30 minutes to fix the nucleic acids. The nylon membrane was then blocked at room temperature with TBST containing 5% skim milk powder for 1 hour. Subsequently, the membrane was incubated overnight at 4°C with an m5C antibody on a shaking platform. The next day, after TBST washing, the membrane was incubated with an HRP-conjugated secondary antibody at room temperature for 1 hour. Signal detection was performed by exposure to ECL chemiluminescent reagent. After stripping the antibody, the membrane was stained with 0.02% methylene blue for 5 minutes, the stained membrane was then scanned as an internal control for nucleic acid loading.

**Cell apoptosis**

Huh7-LR and PLC-LR Cells were seeded into 6-well plates at a density of 1 × 10⁵ cells per well and subjected to respective drug treatments. After incubation for 48 hours, apoptosis was detected using the Annexin V-FITC/PI Apoptosis Detection Kit (KeyGEN Biotech, China). The results were analyzed using an Attune NxT flow cytometer (ThermoFisher, USA) and FlowJo software.

**Immunohistochemistry (IHC) and Scoring**

Formalin-fixed, paraffin-embedded tissue sections were deparaffinized and rehydrated. Antigen retrieval was performed using citrate buffer (pH 6.0) with heat treatment. Endogenous peroxidase activity was blocked with 3% hydrogen peroxide, and non-specific binding sites were blocked by incubation with an appropriate normal serum. The sections were then incubated overnight at 4°C with primary antibodies against [target protein name] (manufacturer, catalog number, dilution ratio). The following day, the sections were incubated with corresponding HRP-conjugated secondary antibodies at room temperature, followed by color development with DAB and counterstaining with hematoxylin. Finally, the sections were dehydrated, cleared, and mounted with neutral gum.

Two pathologists, blinded to the clinicopathological data, independently evaluated all sections. The H-Score, a semi-quantitative scoring system that incorporates both the percentage of positively stained cells and the staining intensity, was calculated using the following formula:H-Score = Σ (Pi × i), where Pi represents the percentage of tumor cells stained at intensity “i”, and “i” denotes the staining intensity (0 = no staining; 1 = weak; 2 = moderate; 3 = strong). The final score ranges from 0 to 300. To minimize inter-observer variability, the scores from the two evaluators were averaged to obtain the final H-Score for each sample. Based on preliminary data analysis in this study, an H-Score ≥ 100 was defined as the high-expression group, while an H-Score < 100 was defined as the low-expression group.

**m5C MeRIP-seq and MeRIP-qPCR**

Target cells were collected and total RNA was extracted using TRIzol. The extracted total RNA was fragmented into 100–200 nt segments, and then the samples were divided into two aliquots. One aliquot of fragmented RNA was incubated overnight at 4°C with anti-m5C antibody in IP buffer. Protein A/G magnetic beads were then added to capture the antibody-RNA complexes. The beads were washed with high-salt buffer to remove nonspecific binding. Subsequently, the RNA was purified by phenol-chloroform extraction and ethanol precipitation, and the purified RNA was eluted. The other aliquot served directly as the Input control. Subsequent library construction and sequencing were outsourced to Jiayin Biotechnology(Shanghai,China). If performing MeRIP-qPCR, the sequencing step was replaced by qPCR at this stage.

**RNA Stability Assay**

After the cells reached the logarithmic growth phase, actinomycin D (5 μg/mL) was added to the experimental group. Cells were rapidly collected at predefined time points (e.g., 0, 2, 4, 8 hours)，total RNA was extracted, followed by RT-qPCR analysis. Finally, GraphPad Prism9.0 was used to generate graphs and calculate the half-life.

**RNA Affinity Chromatography**

50 picomoles of biotinylated RNA fragments, including 50 bp RNA sequences with m5C-modified (ACLY [m5C]) or unmodified (ACLY [C]), were incubated with 2 mg of ganetespib-resistant cell protein extracts and 50 μL of streptavidin beads. Following RNA pull-down assays, the bound proteins were analyzed by Western blotting.

**Immunofluorescence assay**

Huh7-LR and PLC-LR cells were seeded onto confocal dishes and cultured overnight (1×10⁵ cells per well). The cells were fixed with 4% paraformaldehyde at room temperature for 15 minutes, then washed three times with PBS. Subsequently, cell membranes were permeabilized with 0.5% Triton X-100 for 10 minutes, followed by three washes with PBS. After blocking with 5% goat serum at room temperature for 1 hour, the samples were incubated with anti-H3K27ac primary antibody at 4°C overnight. The following day, fluorescently labeled secondary antibody (Alexa Fluor 488) was added dropwise, and the samples were incubated in the dark at room temperature for 1 hour. After three washes with PBS, nuclei were stained with DAPI, and images were captured and saved.

**Cleavage Under Targets and Tagmentation(CUT&Tag)**

The samples were crosslinked with 1% formaldehyde at room temperature for 10 minutes, followed by quenching with glycine. The samples were then treated with permeabilization buffer containing 0.01% digitonin for 10 minutes. Primary antibody (anti-H3K27) was added and incubated overnight at 4°C, followed by incubation with secondary antibody at room temperature for 1 hour. Unbound antibodies were removed by washing. Subsequently, Protein A-Tn5 fusion protein was added and incubated at room temperature for 1 hour. Tn5 enzymatic activity was then activated by adding Mg²⁺, and the mixture was incubated at 37°C for 1 hour. Finally, DNA fragments were released by digestion with proteinase K and SDS at 65°C for 1 hour. The DNA was then PCR-amplified and subjected to paired-end sequencing on an Illumina platform.

**Assay for Transposase Accessible Chromatin(ATAC)**

Fresh viable cells (recommended ≥50,000 cells, viability >80%) were collected and treated with pre-cooled lysis buffer containing 0.1% NP-40 for 10 minutes to lyse the plasma membrane. The nuclei were then isolated by centrifugation. The nuclei were mixed with Tn5 transposase complexed with sequencing adaptors and incubated at 37°C for 30 minutes. Tn5 specifically cleaved open chromatin regions and simultaneously ligated sequencing adaptors. The reaction was subsequentlyterminated with SDS. Following digestion with proteinase K, the DNA fragments were purified by phenol-chloroform extraction. PCR amplification was performed 5–12 cycles using index primers. Magnetic bead-based size selection was carried out to remove fragments larger than 1,000 bp and mitochondrial DNA contamination. Paired-end sequencing was conducted on an Illumina platform. The raw sequencing data underwent quality control, alignment to the reference genome, and peak calling using tools such as MACS2 to identify open chromatin regions.

**Chromatin immunoprecipitation(ChIP)-qPCR**

ChIP assays were performed using an anti-H3K27ac antibody according to the manufacturer's protocol(Abcam). Enrichment fold-changes were calculated by quantitative real-time PCR (RT-qPCR) and expressed as the percentage of input chromatin (% input).

**HCC organoid culture**

Lenvatinib-resistant organoid models were established using HCC specimens from patients who had not received neoadjuvant therapy. HCC organoids were subcutaneously injected into immunodeficient mice. Starting 15 days post-inoculation, the mice were treated with 10 mg/kg lenvatinib until day 30. Tumor size was monitored throughout the xenotransplantation process. Lenvatinib-resistant tumors were selected for subsequent model construction, and this selection process was repeated twice.

Fresh HCC tissue samples were washed with PBS and mechanically minced. The tissue fragments were then enzymatically digested using a solution containing collagenase to obtain a single-cell suspension. The suspension was subsequently purified through filtration, centrifugation, and red blood cell lysis. The resulting single cells were resuspended in an appropriate volume of complete organoid culture medium and thoroughly mixed on ice with pre-cooled Matrigel at a 1:2 volume ratio.

The mixture was then plated at a density of 10,000 cells per 50 µL aliquot in the center of wells within a 24-well culture plate. The plate was incubated at 37°C for 15 minutes to allow complete polymerization and solidification of the Matrigel. Following this, 500 µL of complete organoid culture medium was gently added along the wall of each well. The plate was subsequently placed in a 37°C incubator with 5% CO₂. Organoid growth and morphology were regularly observed under a microscope. One week after successful model establishment, the organoids were subjected to a 4-day drug intervention with BMS-303141 (10 µmol), lenvatinib (10 µmol), or a combination of both drugs.

**Supplementary Figures**


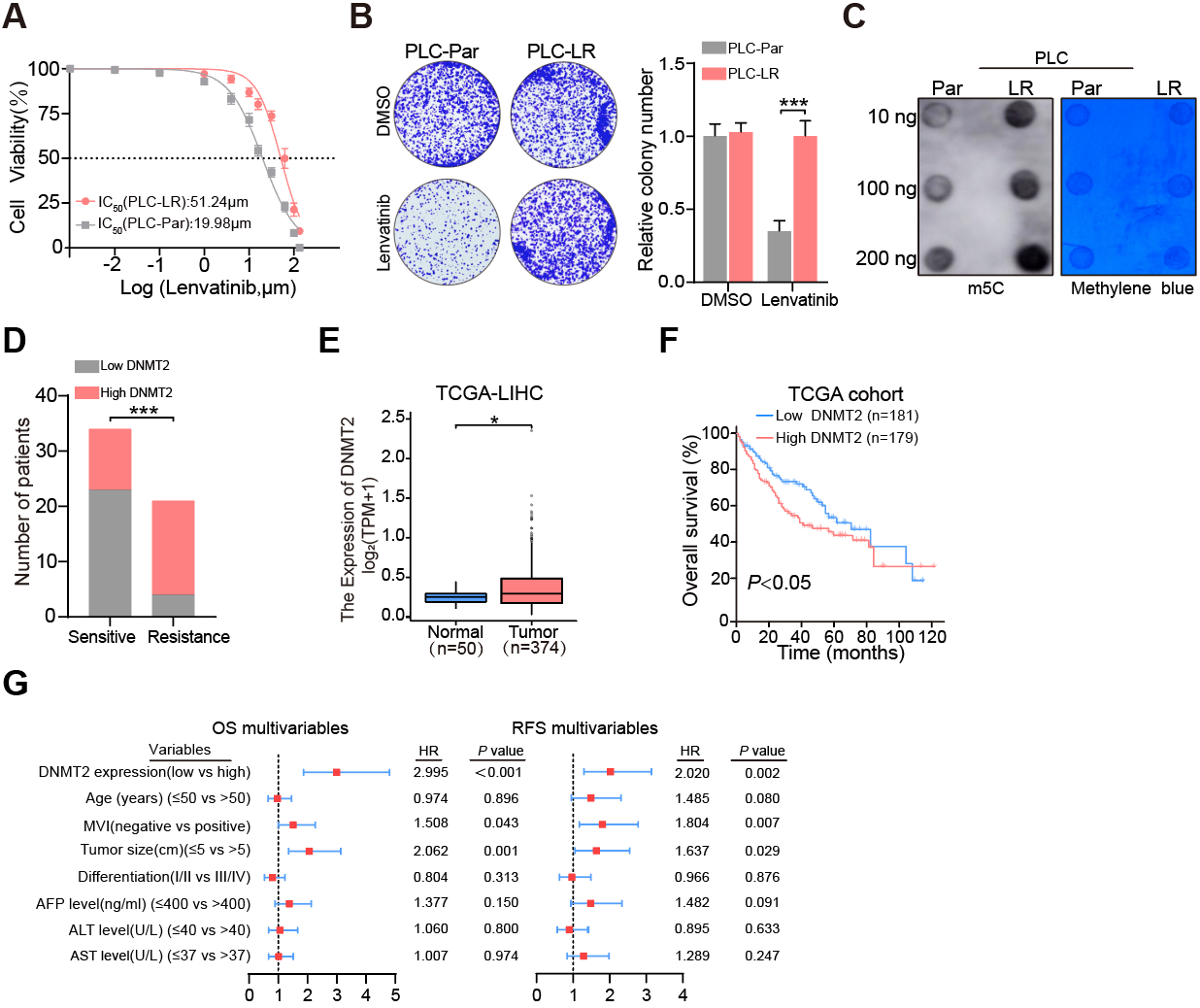


**Figure S1 m5C hypermethylation and DNMT2 overexpression are associated with lenvatinib resistance in HCC**

**(A)** IC₅₀ curves of lenvatinib in parental PLC vs. lenvatinib-resistant PLC cells. **(B)** Colony formation assay in parental and lenvatinib-resistant PLC cells post-lenvatinib treatment (30 µM). **(C)** Dot blot quantification of global m5C modification levels in parental vs. lenvatinib-resistant cells. **(D)** Correlation between DNMT2 expression and lenvatinib treatment response. **(E)** Relative DNMT2 expression in TCGA-LIHC cohort. **(F)** Survival analysis of TCGA-LIHC patients stratified by DNMT2 expression. **(G)** Multivariate Cox analysis of OS and RFS clinical prognostic parameters. **P* < 0.05; ***P* < 0.01; ****P* < 0.001.


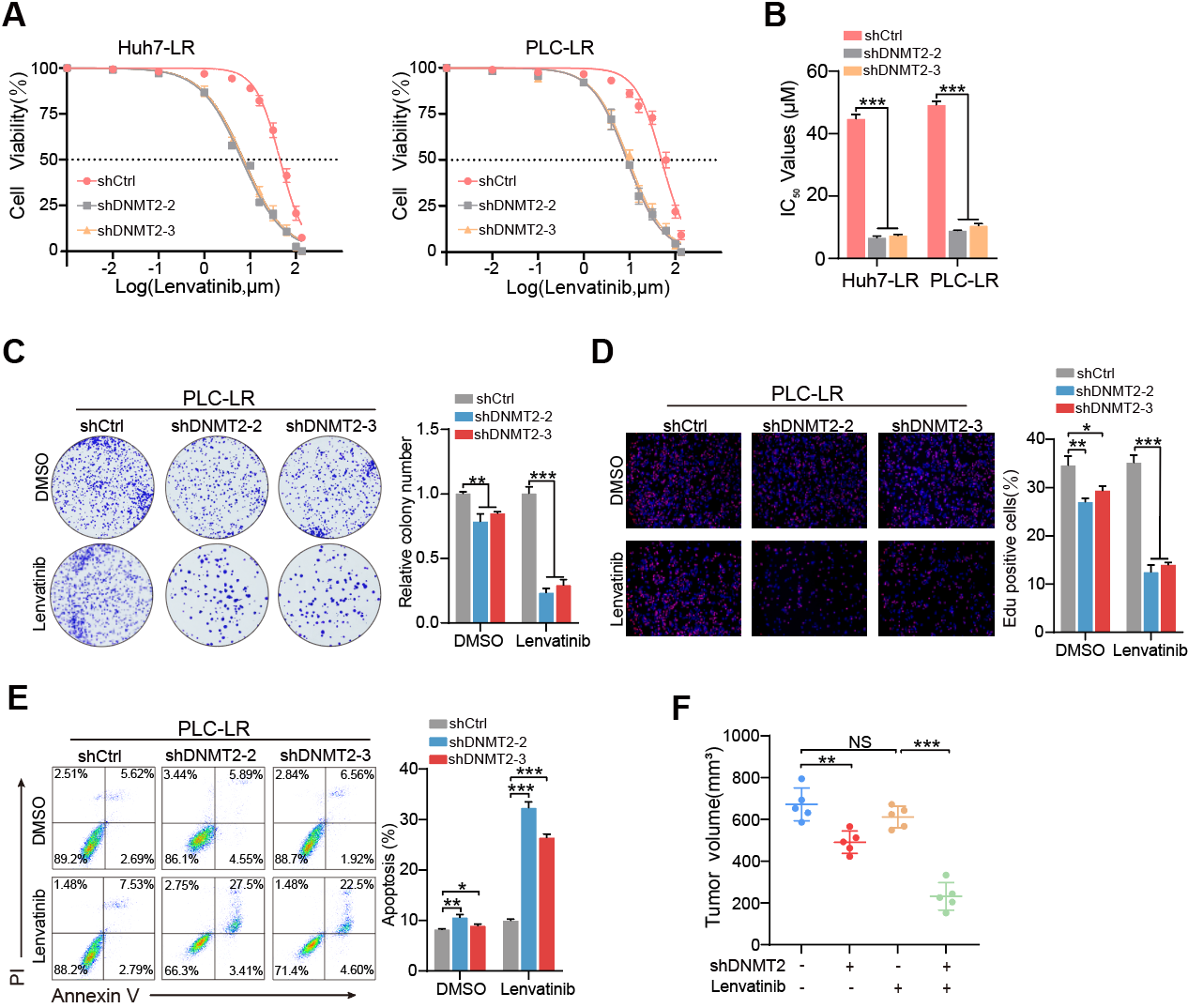


**Figure S2 DNMT2 modulats the therapeutic sensitivity of HCC cells to lenvatinib**

**(A–B)** IC_50_ curves of parental and lenvatinib-resistant cells following DNMT2 knockdown. **(C–D)** Colony formation and EdU assays evaluating proliferative changes in PLC-LR cells after DNMT2 knockdown. **(E)** Flow cytometry analysis of Annexin V and PI staining in PLC-LR cells. **(F)** Tumor volume differences among the four groups in mice. **P* < 0.05; ***P* < 0.01; ****P* < 0.001.


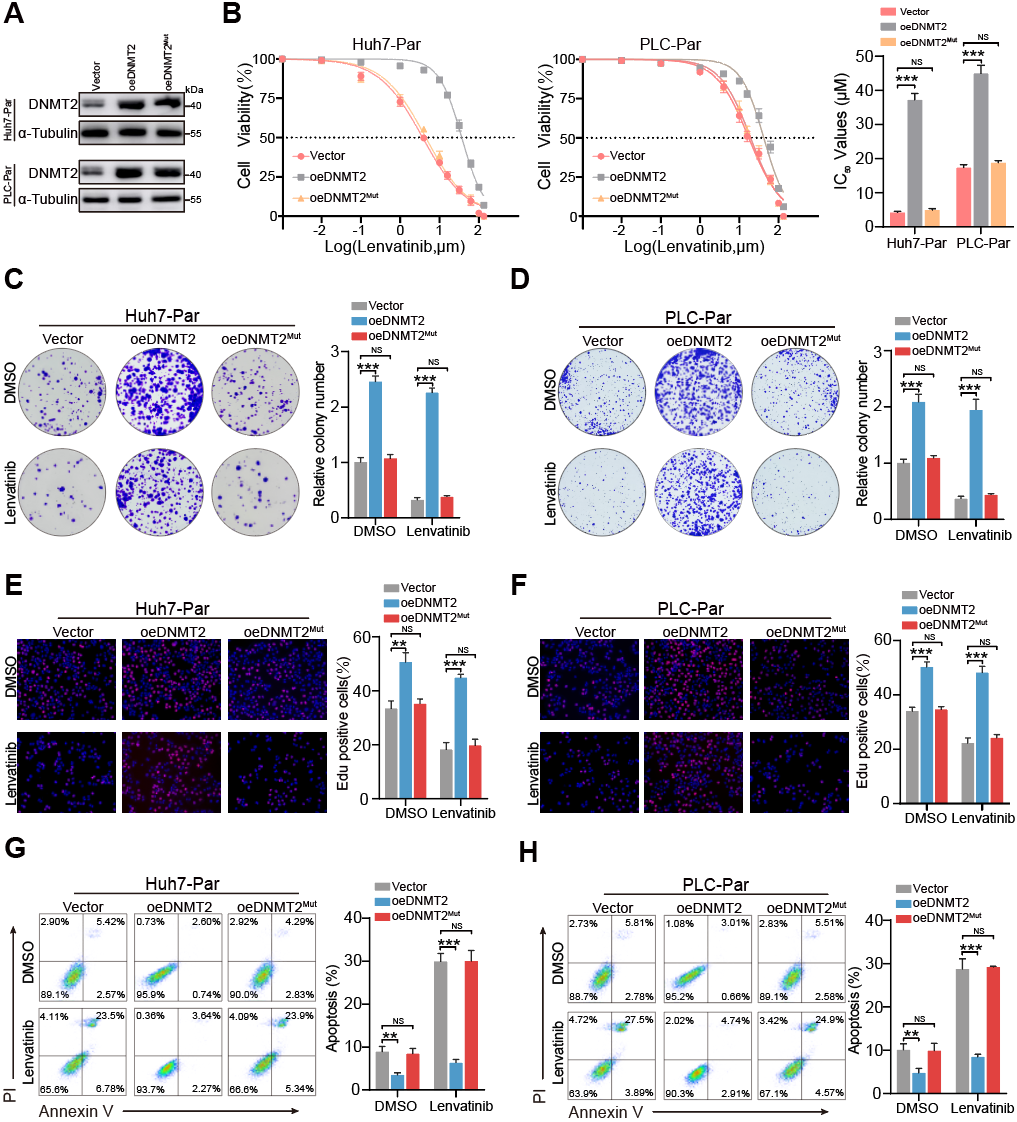


**Figure S3 DNMT2 modulats the therapeutic sensitivity of HCC cells to lenvatinib**

**(A)** Verification of transfection efficiency following DNMT2 overexpression of wild-type DNMT2 or catalytic mutant DNMT2. **(B)** IC_50_ curves of parental and lenvatinib-resistant cells.

**(C-F)** Colony formation and EdU assays evaluating proliferative changes in HCC cells after overexpression of wild-type DNMT2 or catalytic mutant DNMT2. **(G-H)** Flow cytometry analysis of Annexin V and PI staining in HCC cells. **P* < 0.05; ***P* < 0.01; ****P* < 0.001.


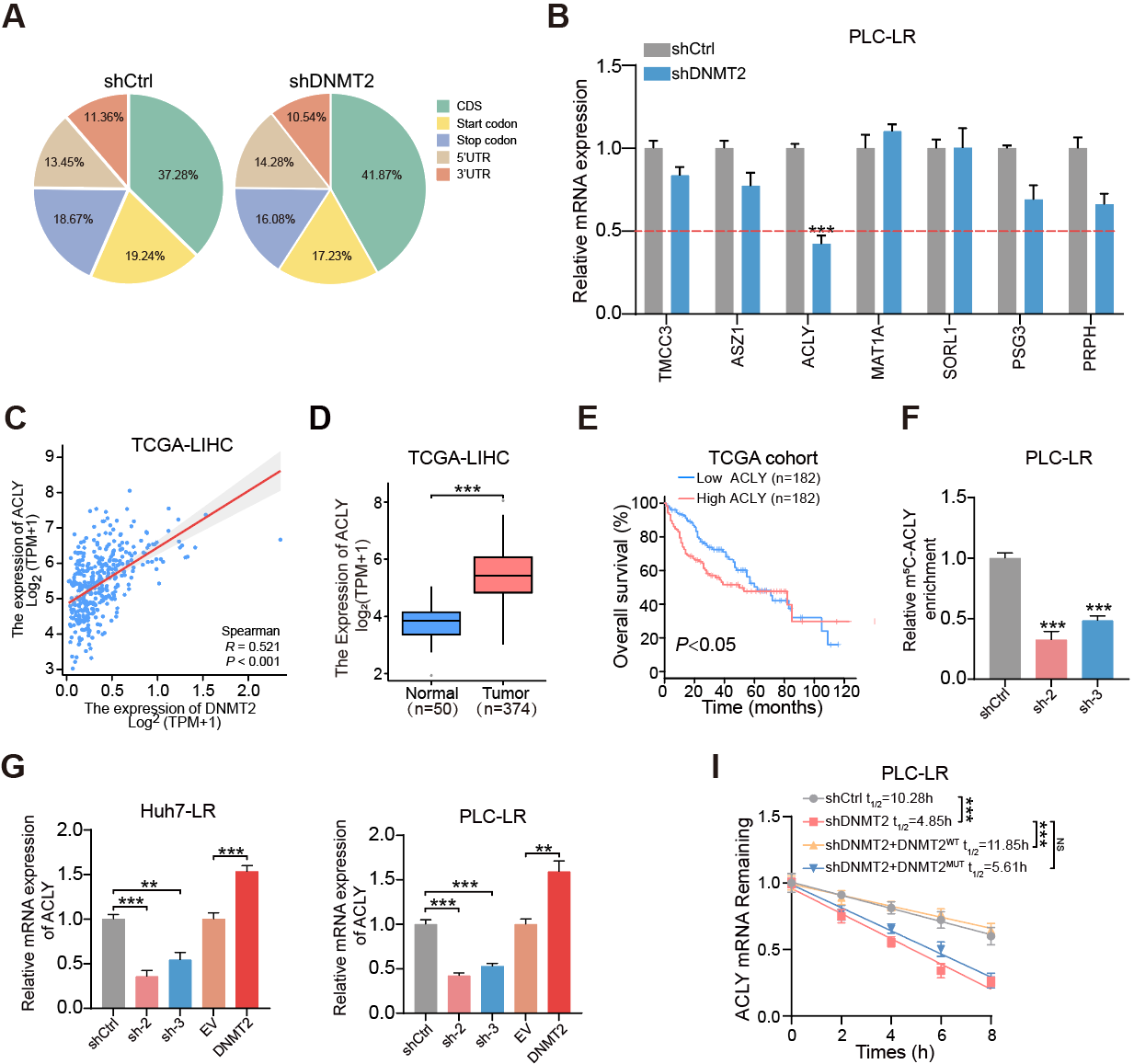

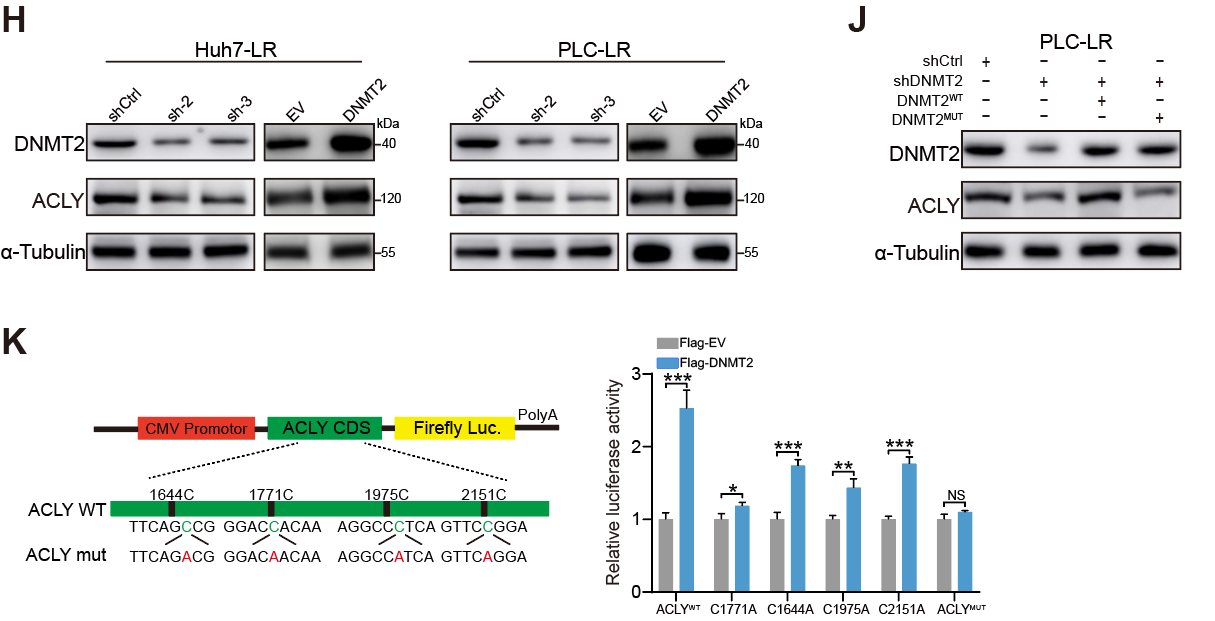


**Figure S4** **DNMT2 enhances the stability of ACLY mRNA through m5C-YBX1-dependent methylation modification**

**(A)** m5C modification levels in different gene regions in control and shDNMT2 cells. **(B)** Expression changes of seven candidate target genes in PLC-LR cells after shDNMT2 treatment.

**(C)** Correlation analysis between DNMT2 and ACLY mRNA expression levels in the TCGA-LIHC database. **(D–E)** The expression levels of ACLY in the TCGA-LIHC database and the correlation between ACLY expression levels and patient survival prognosis. **(F)** MeRIP-qPCR evaluating ACLY m5C modification abundance in PLC-LR cells after DNMT2 knockdown. **(G-H)** RT-qPCR and WB assessing the effects of DNMT2 knockdown or overexpression on ACLY expression. **(I)** Analysis of ACLY mRNA half-life in PLC-LR cells treated with the different vectors. **(J)** WB analysis of ACLY protein expression in PLC-LR cells treated with the different vectors. **(K)** The m5C sites screening on ACLY. **P* < 0.05; ***P* < 0.01; ****P* < 0.001.


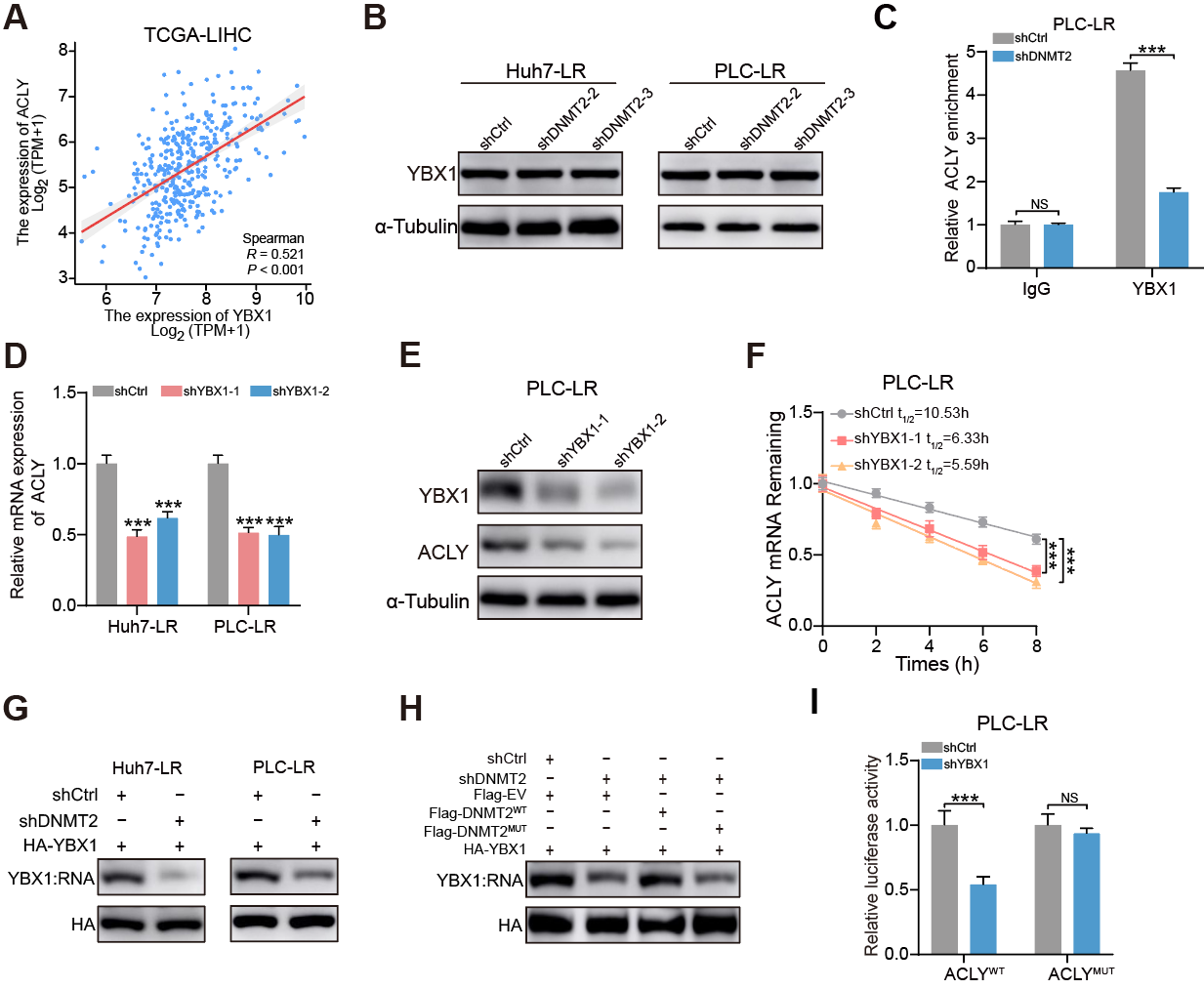


**Figure S5 DNMT2 enhances the stability of ACLY mRNA through m5C-YBX1-dependent methylation modification**

**(A)** Correlation analysis of YBX1 and ACLY mRNA expression levels in the TCGA-LIHC database. **(B)** Impact of DNMT2 knockdown on YBX1 protein expression in resistant cells. **(C)** RIP-qPCR analysis showing the difference in YBX1 binding to ACLY RNA between control and DNMT2 knockdown PLC-LR cells. **(D)** RT-qPCR evaluating the effect of YBX1 downregulation on ACLY expression in resistant cells. **(E–F)**. Effects of YBX1 knockdown on ACLY protein levels and RNA stability in PLC-LR cells. **(G)** Observation of YBX1-RNA interaction capability after DNMT2 knockdown in Huh7-LR and PLC-LR cells. **(H)** Observation of the interaction ability of YBX1 - RNA after transfection with different vectors. **(I)** Relative luciferase activity of ACLY^WT^ and ACLY^MUT^ reporter vectors in control or YBX1 knockdown PLC-LR cells. **P* < 0.05; ***P* < 0.01; ****P* < 0.001.


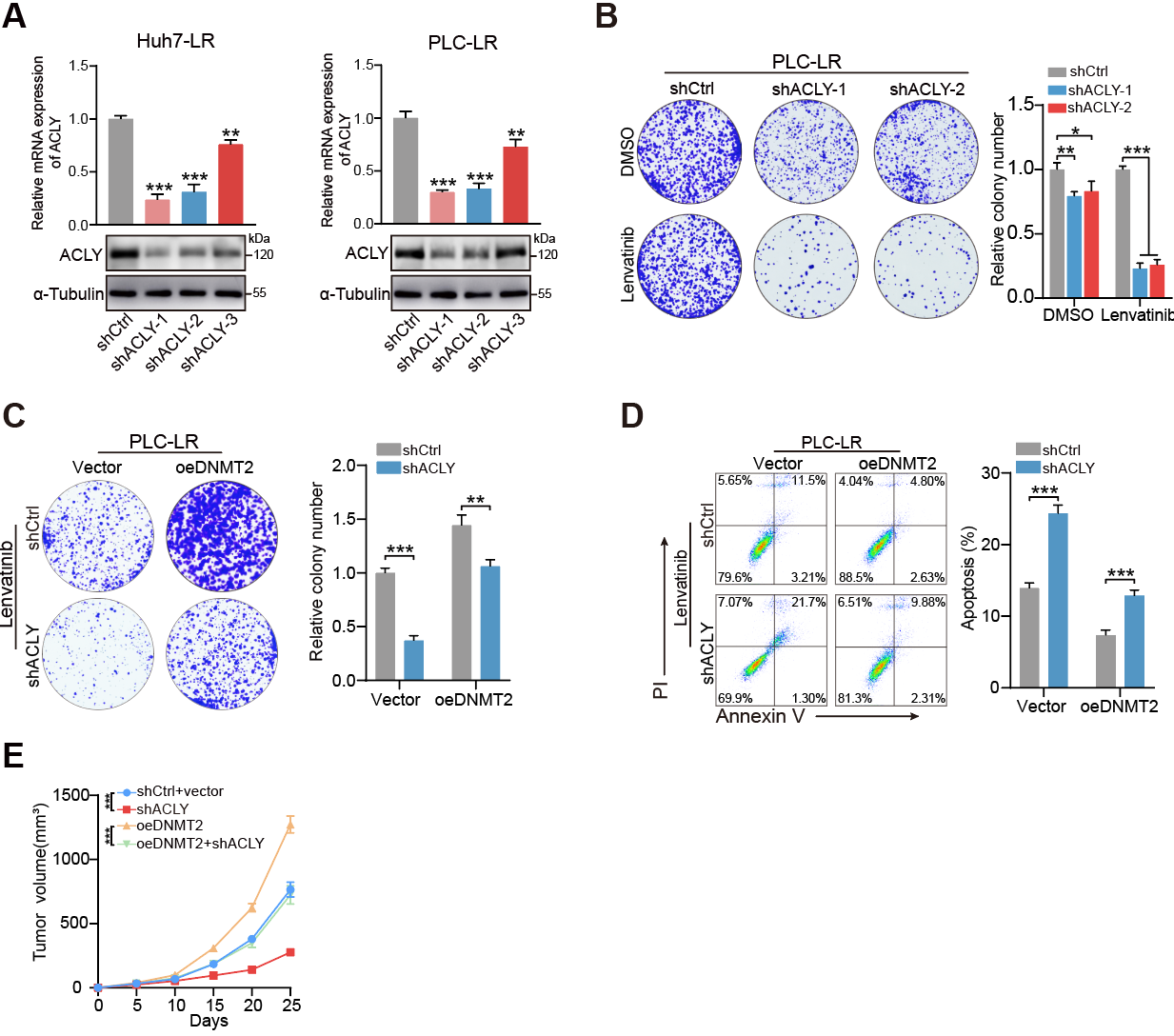


**Figure S6 The DNMT2/ACLY regulatory axis influences lenvatinib resistance in hepatocellular carcinoma**

**(A)** Verification of transfection efficiency following ACLY knockdown in Lenvatinib-resistant cells. **(B)** Colony formation assessing changes in proliferation of PLC-LR cells after ACLY knockdown. **(C)** Colony formation conducted in PLC-LR cells with DNMT2 overexpression or ACLY knockdown. **(D)** Flow cytometry analysis of Annexin V and PI staining in PLC-LR cells with DNMT2 overexpression or ACLY knockdown. **(E)** The subcutaneous tumor growth curves following DNMT2 overexpression or ACLY knockdown. **P* < 0.05; ***P* < 0.01; ****P* < 0.001.


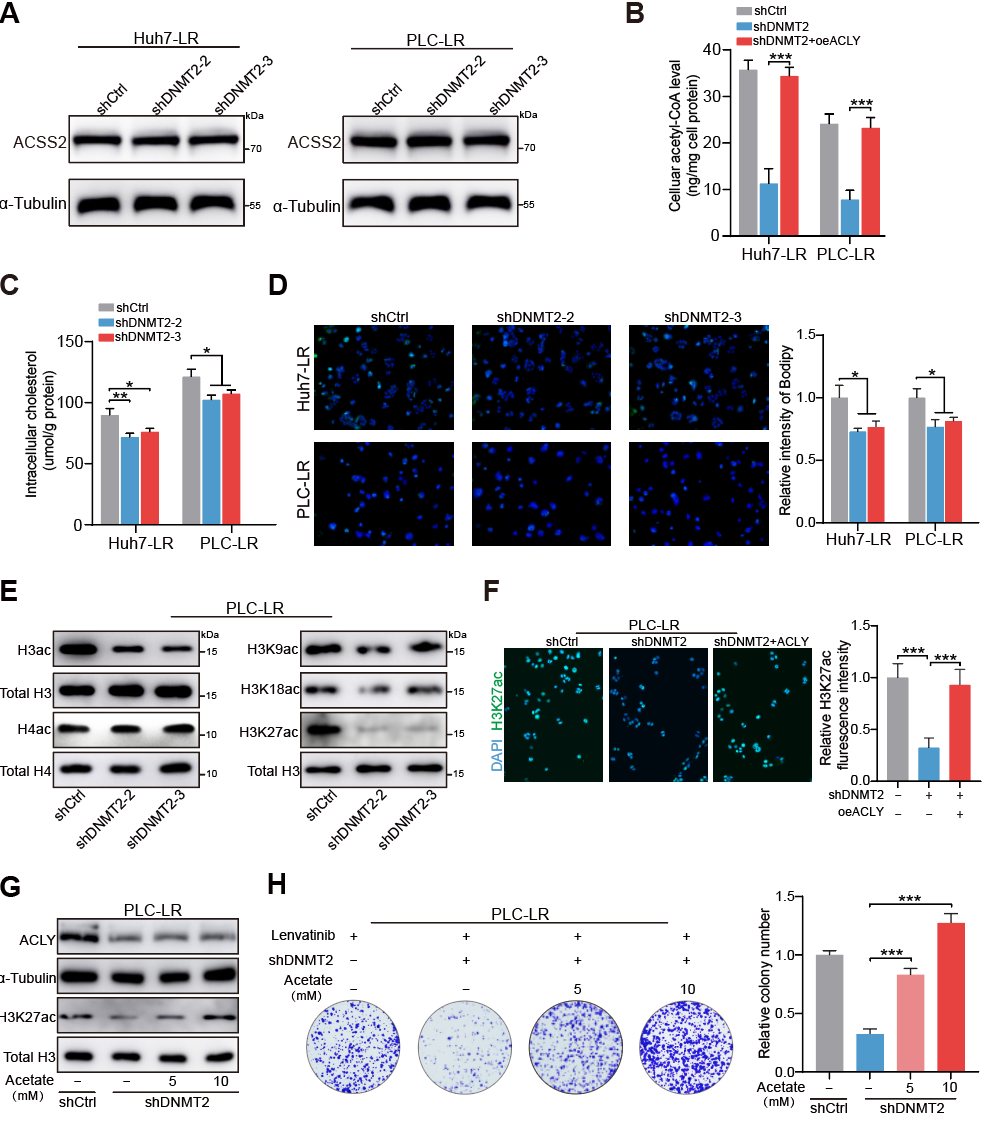


**Figure S7 The DNMT2/ACLY axis promotes lenvatinib resistance by modulating histone acetylation**

**(A)** Impact of DNMT2 knockdown on ACSS2 protein expression in drug-resistant cells. **(B)** Effects of DNMT2 knockdown or ACLY overexpression on acetyl-CoA levels in resistant cells.

**(C–D)** Effects of DNMT2 knockdown on lipid levels within resistant cells. **(E)** Levels of histone H3 and H4 acetylation in PLC-LR cells following DNMT2 knockdown. **(F)** Effects of DNMT2 knockdown or ACLY overexpression on H3K27ac staining(scale bar, 100 μm). **(G–H)** Impact of DNMT2 knockdown or exogenous acetate addition on H3K27 acetylation and cell growth activity in Huh7-LR cells. **P* < 0.05; ***P* < 0.01; ****P* < 0.001.


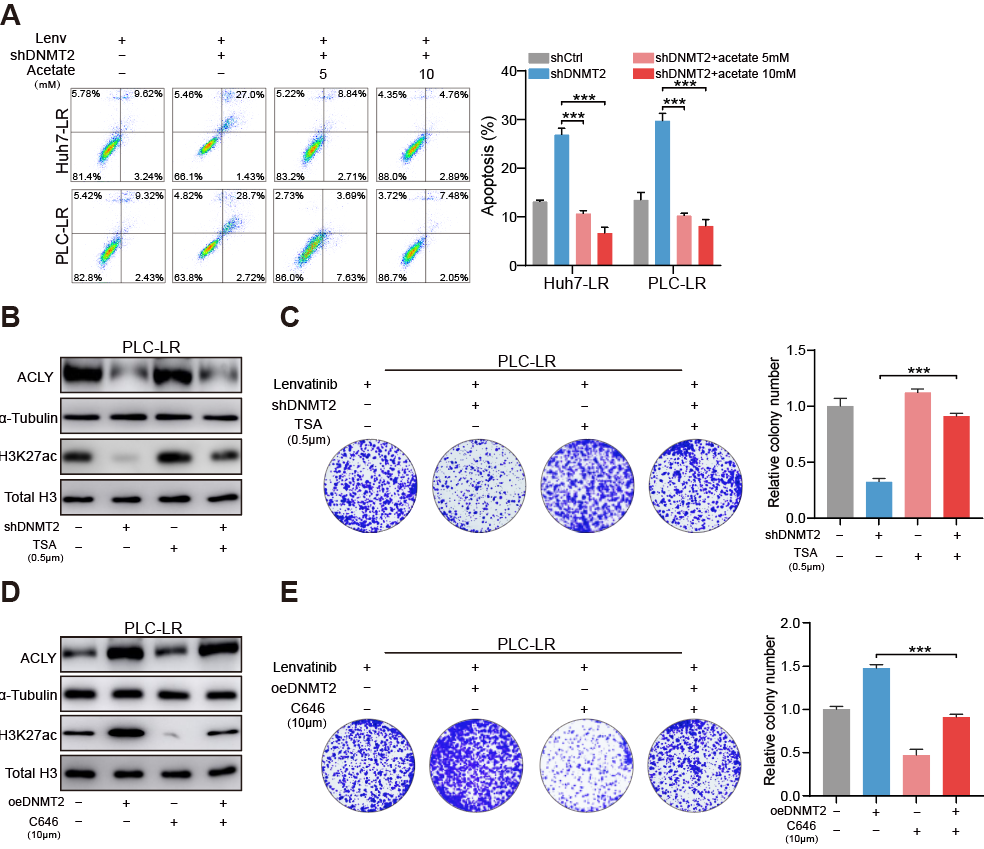


**Figure S8 The DNMT2/ACLY axis promotes lenvatinib resistance by modulating histone acetylation**

**(A)** Effect of DNMT2 knockdown or exogenous acetate addition on apoptosis levels in resistant cells. **(B–C)** Effects of DNMT2 knockdown or TSA treatment on H3K27 acetylation and cell viability in PLC-LR cells. **(D–E)** Impact of DNMT2 overexpression or C646 treatment on H3K27 acetylation and cell viability in PLC-LR cells. **P* < 0.05; ***P* < 0.01; ****P* < 0.001.


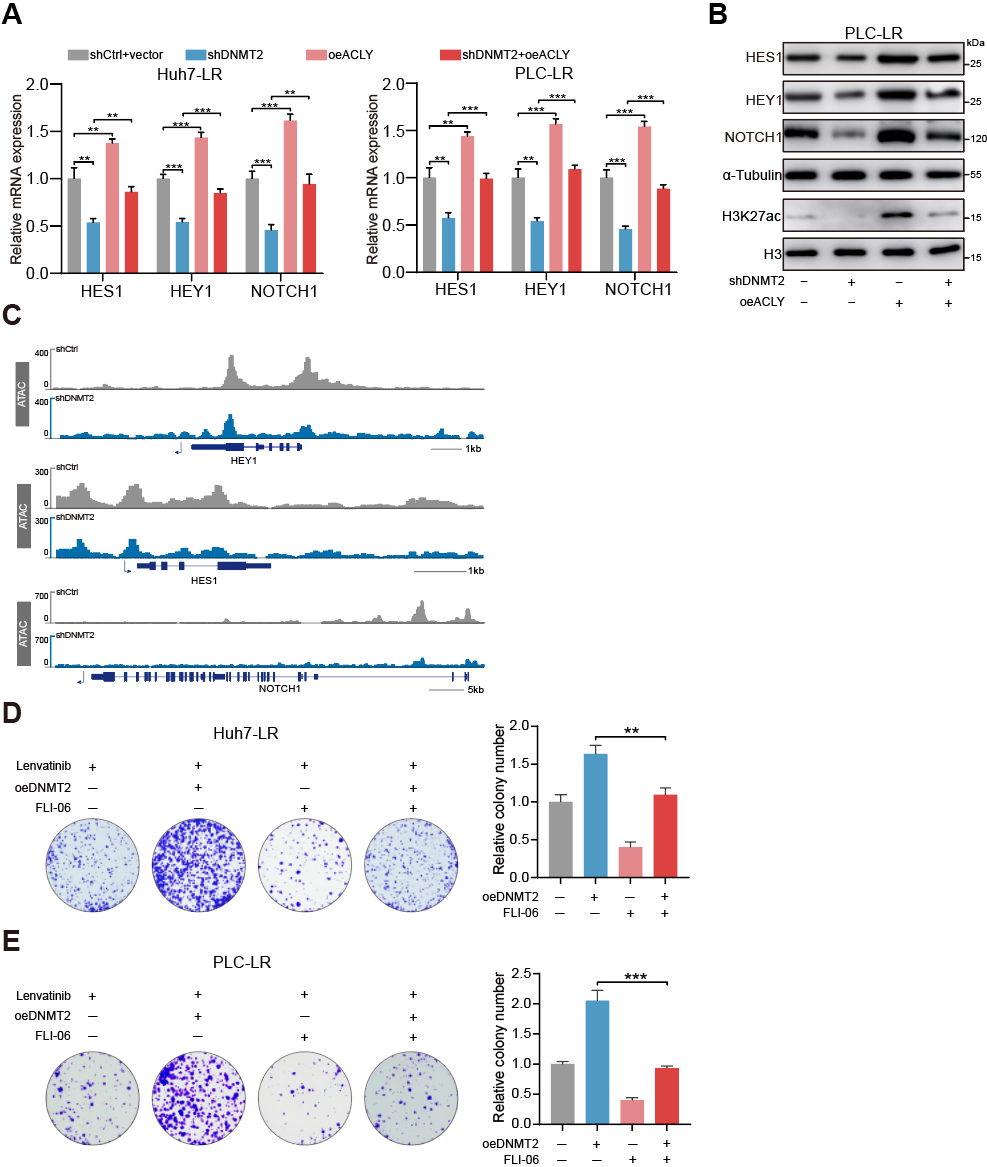


**Figure S9 H3K27 acetylation epigenetic remodeling activates the transcription of the Notch signaling pathway**

**(A)** RT-qPCR detection of expression changes in NOTCH1, HES1, and HEY1 mRNA in resistant cells following DNMT2 knockdown or ACLY overexpression. **(B)** Effects of DNMT2 knockdown or ACLY overexpression on protein levels of NOTCH1, HES1, and HEY in PLC-LR cells. **(C)** Visualization of chromatin accessibility at the promoters of NOTCH1, HES1, and HEY1 using ATAC-seq following DNMT2 knockdown. **(D–E)** Effects of DNMT2 overexpression or FLI-06 on the proliferation capacity of resistant cells. **P* < 0.05; ***P* < 0.01; ****P* < 0.001.


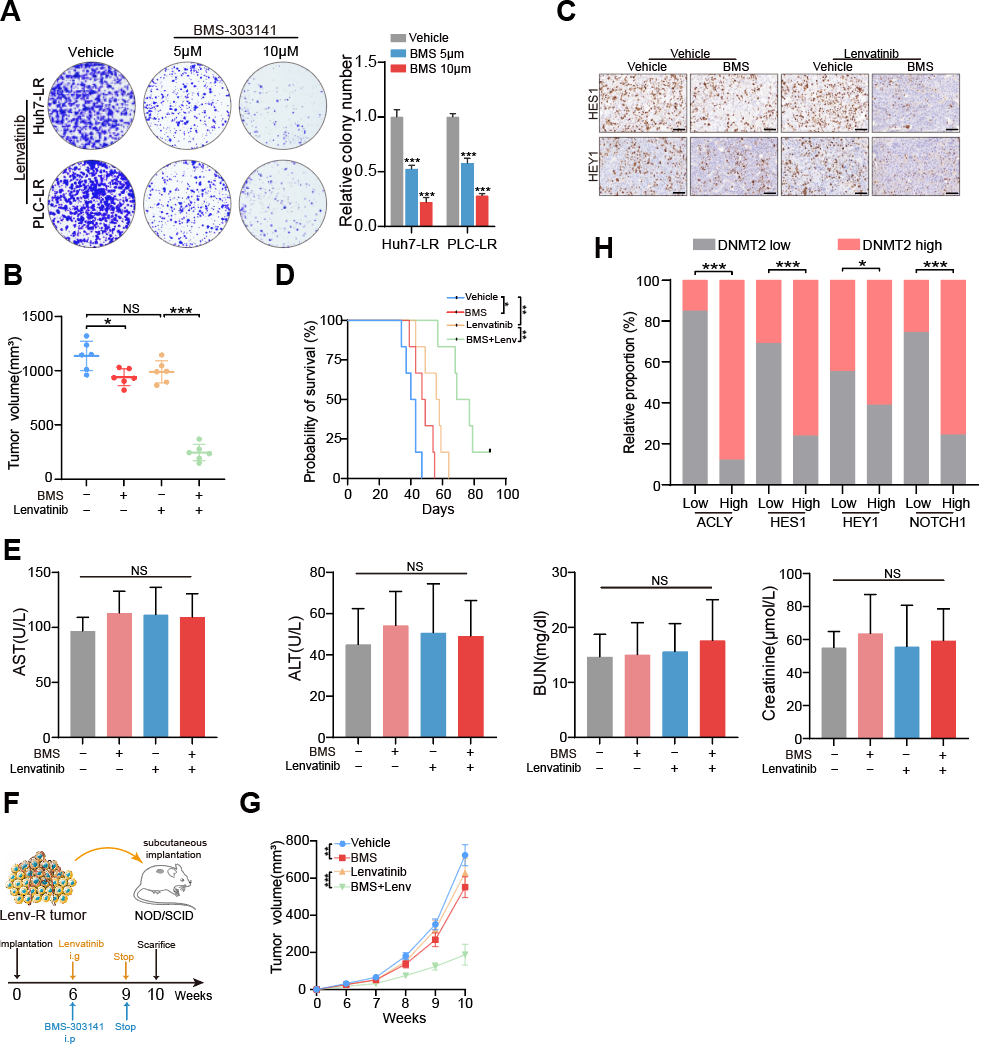


**Figure S10 The ACLY inhibitor enhances the therapeutic efficacy of lenvatinib in multiple preclinical HCC models**

**(A)** Effect of combined BMS-303141 and lenvatinib on colony formation ability in resistant cells. **(B)** Influence of combined treatment on tumor volume in the orthotopic liver cancer model in mice. **(C)** Immunohistochemical detection of HES1 and HEY in tissue samples from the orthotopic liver cancer model(scale bar, 50 μm). **(D)** Impact of combined BMS-303141 and lenvatinib on survival rates in mice bearing spontaneous liver tumors. **(E)** Effect of combined BMS-303141 and lenvatinib on liver and kidney functions in treated mice. **(F–G)** Schematic diagram of the construction process of lenvatinib-resistant PDX models and tumor growth curves of PDX models. **(H)** Quantitative analysis of the correlations between DNMT2 expression and the expression levels of ACLY, HES1, HEY1, and NOTCH1. **P* < 0.05; ***P* < 0.01; ****P* < 0.001.

**
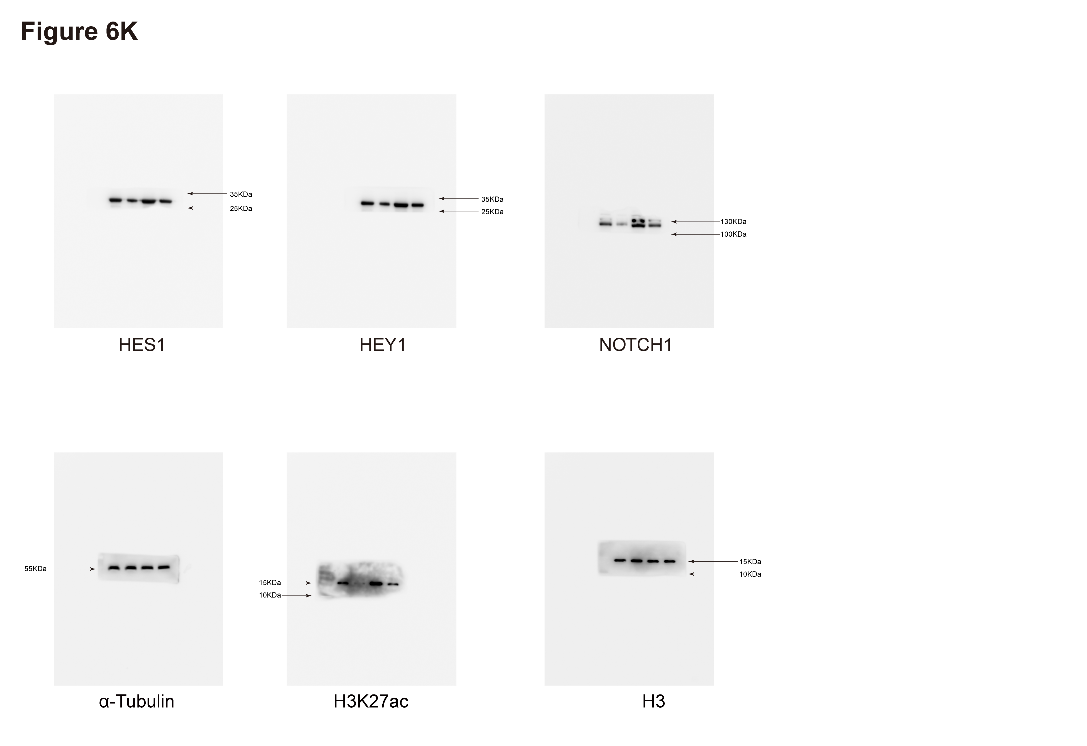
**

**
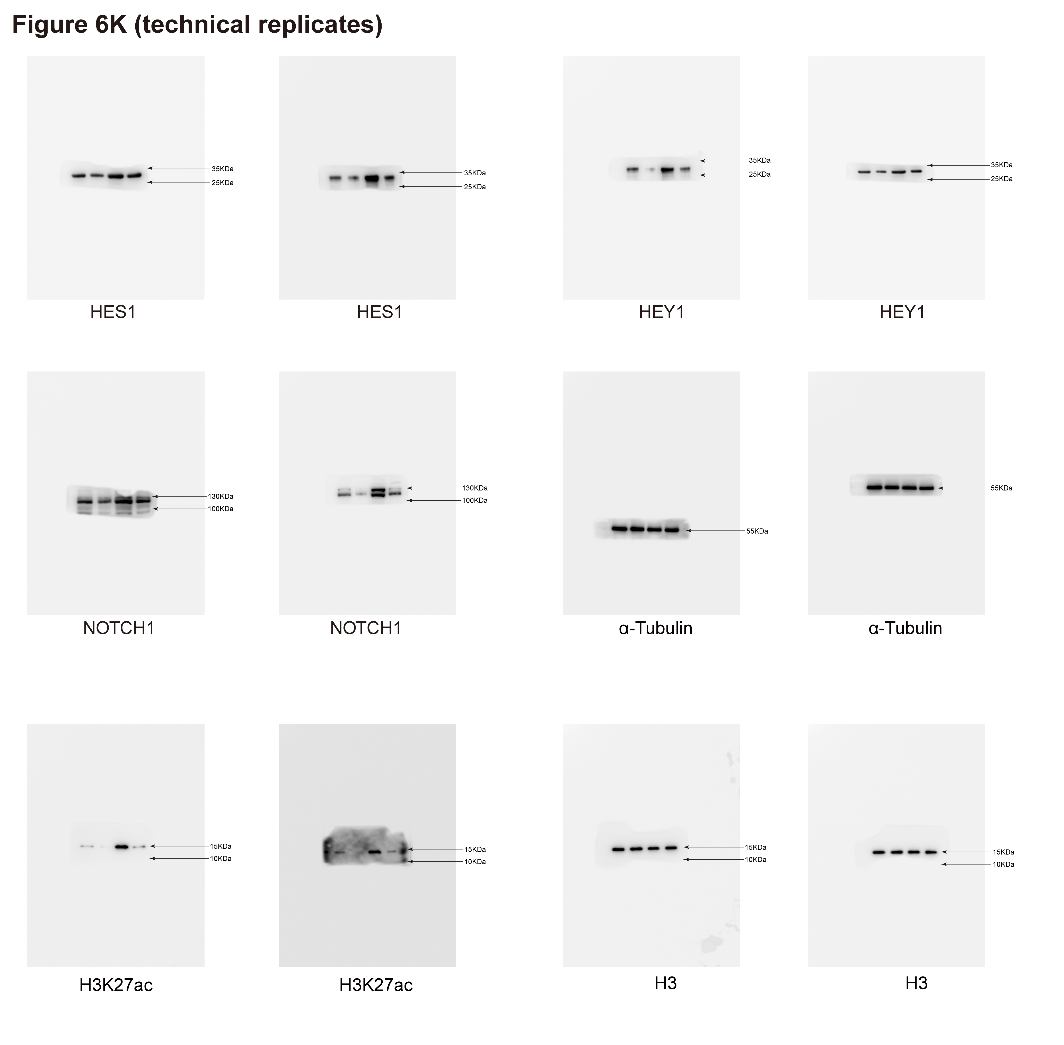
**

**Figure S11 The raw data of Western Blot images**

**Supplemental Table-1 Clinicopathologic characteristics of patients treated by Lenvatinib in Zhongshan cohort1.**

|  | | | | |  |
| --- | --- | --- | --- | --- | --- |
| **Variable** | **Number of patients** | | |  |  |
|  | Sensitive (n=34) | | Resistance (n=21) | |  |
|  |  |  |  |  |  |
| Age (y) |  |  |  |  |  |
| ≤50 | 9 | | 5 | |  |
| >50 | 25 | | 16 | |  |
| Gender |  | |  |  |  |
| Female | 12 | | 10 | |  |
| Male | 22 | | 11 | |  |
| Liver cirrhosis |  | |  |  |  |
| No | 7 | | 4 | |  |
| Yes | 27 | | 17 | |  |
| Hepatitis history |  | |  |  |  |
| Negative | 5 | | 3 | |  |
| Positive | 29 | | 18 | |  |
| AFP (ng/ml) |  | |  |  |  |
| ≤400 | 8 | | 7 | |  |
| >400 | 26 | | 14 | |  |
| TNM stage |  | |  |  |  |
| I-II | 0 | | 0 | |  |
| III-IV | 34 | | 21 | |  |
|  | | | |  |  |

**Supplemental Table-2 Relationships between DNMT2 and clinicopathological characteristics in the Zhongshan cohort2**

| **Variables** | **Total**  **（n=173）** | **DNMT2 low**  **（n=69）** | **DNMT2 high**  **（n=104）** | **χ2** | ***P*** |
| --- | --- | --- | --- | --- | --- |
| Age |  |  |  | 1.674 | 0.196 |
| ≤50 | 73(42.2) | 25 | 48 |  |  |
| >50 | 100(57.8) | 44 | 56 |  |  |
| AFP |  |  |  | 1.913 | 0.167 |
| ≤400 | 62(35.8) | 29 | 33 |  |  |
| >400 | 111(64.2) | 40 | 71 |  |  |
| CEA |  |  |  | 0.230 | 0.631 |
| ≤5 | 160(92.5) | 63 | 97 |  |  |
| >5 | 13(7.5) | 6 | 7 |  |  |
| CA199 |  |  |  | 0.993 | 0.319 |
| ≤36 | 131(75.7) | 55 | 76 |  |  |
| >36 | 42(24.3) | 14 | 28 |  |  |
| Cirrhosis |  |  |  | 2.610 | 0.106 |
| No | 28(16.2) | 15 | 13 |  |  |
| Yes | 145(83.8) | 54 | 91 |  |  |
| Tumor size |  |  |  |  |  |
| ≤5 | 82(47.4) | 40 | 42 | 5.145 | 0.023 |
| >5 | 91(52.6) | 29 | 62 |  |  |
| Differentiation |  |  |  |  |  |
| I/II | 110(63.6) | 49 | 61 | 2.737 | 0.098 |
| III/IV | 63(36.4) | 20 | 43 |  |  |
| Child grade |  |  |  |  |  |
| A | 163(94.2) | 64 | 99 | 0.453 | 0.501 |
| B/C | 10(5.8) | 5 | 5 |  |  |
| MVI |  |  |  |  |  |
| Negative | 99(57.2) | 48 | 51 | 7.140 | 0.008 |
| Positive | 74(42.8) | 21 | 53 |  |  |
| BCLC stage |  |  |  |  |  |
| 0/A | 85(49.1) | 39 | 46 | 2.507 | 0.113 |
| B/C | 88(50.9) | 30 | 58 |  |  |
| ALT |  |  |  |  |  |
| ≤40 | 100(57.8) | 35 | 65 | 2.358 | 0.125 |
| >40 | 73(42.2) | 34 | 39 |  |  |
| AST |  |  |  |  |  |
| ≤37 | 124(71.7) | 55 | 69 | 3.649 | 0.056 |
| >37 | 49(28.3) | 14 | 35 |  |  |

**Supplemental Table-3 Primers sequences used for RT-qPCR**

| **Gene** |  | **Sequence** |
| --- | --- | --- |
| DNMT2 | Forward | 5′- CACAAGTGGTGGCTGCCATTGA -3′′ |
|  | Reverse | 5′- GTCAAACTCTTCGAGTGTAATGCC -3′ |
| ACLY | Forward | 5′- GCTCTGCCTATGACAGCACCAT -3′ |
|  | Reverse | 5′- GTCCGATGATGGTCACTCCCTT -3′ |
| YBX1 | Forward | 5′- GCAGGAGAACAAGGTAGACCAG -3′  CACGCTTTCCAAGGCAGAAA  CACGCTTTCCAAGGCAGAAA  - 3′ |
|  | Reverse | 5′- CTTCATTGCCGTCCTCTCTAGG -3′  TGAAGTCGTGGGAGCAAGTC  TGAAGTCGTGGGAGCAAGTC  -3′ |
| NOTCH1 | Forward | 5′-GGTGAACTGCTCTGAGGAGATC -3′ |
|  | Reverse | 5′-GGATTGCAGTCGTCCACGTTGA -3′  GCGTGCCGGTCATAGAAGAA  GCGTGCCGGTCATAGAAGAA  GCGTGCCGGTCATAGAAGAA  GCGTGCCGGTCATAGAAGAA |
| HES1 | Forward | 5′- GGAAATGACAGTGAAGCACCTCC -3′ |
|  | Reverse | 5′- GAAGCGGGTCACCTCGTTCATG -3′ |
| HEY1 | Forward | 5′- TGTCTGAGCTGAGAAGGCTGGT -3′ |
|  | Reverse | 5′- TTCAGGTGATCCACGGTCATCTG -3′ |
| TMCC3 | Forward | 5′-TGAGACAGCCAACCTGAAGCAG -3′ |
|  | Reverse | 5′- ATGCGAGTCTGGCAGGATTCCA -3′ |
| ASZ1 | Forward | 5′- GCTAGTGTTGCCAATGCAGAGC -3′ |
|  | Reverse | 5′- CTGTTCCTCTGAGCCATGAGCA -3′ |
| MAT1A | Forward | 5′- GCCAAGTCTCTGGTGAAAGCAG -3′ |
|  | Reverse | 5′- CTGTCTTCTGAGAGGTTCCGTAG -3′ |
| SORL1 | Forward | 5′- GAACACCTGTCTTCGCAACCAG -3′ |
|  | Reverse | 5′- TGTCCAGGTCACAGATGGTGGT -3′ |
| PSG3 | Forward | 5′- CGTAAAGCGAGGTGATGGGACT -3′ |
|  | Reverse | 5′- AAGCTCACAGCCTCCATGTCCT -3′ |
| PRPH | Forward | 5′- CAAGCAGGAGATGAACGAGTCC -3′ |
|  | Reverse | 5′- TCCAGCTCTCTCAACTGCCTGA -3′ |
| GAPDH | Forward | 5′- GTCTCCTCTGACTTCAACAGCG -3′ |
|  | Reverse | 5′- ACCACCCTGTTGCTGTAGCCAA -3′ |

**Supplemental Table-4 Targeted sequences of shRNAs or siRNAs and probe sequences of ACLY used in this study**

| shRNA | Sequence |
| --- | --- |
| shDNMT2-1 CACCAGATCGTGCCCGAGGGCGAG 3’ | 5'- AGCGTTTATAACACCATTATT -3' |
| shDNMT2-2 | 5'- CCAAAGTCATTGCTGCGATAT -3' |
| shDNMT2-3 | 5'- ATGTCAACACTGTCGCTAATG -3'  -3'  -3' |
| shACLY-1 | 5'- GCTTCATCTCTGGTCTATT -3' |
| shACLY-2 | 5'- GCTGAATACCGAGGACATT -3' |
| shACLY-3 | 5'- CTCAAGATACTATACATTT -3' |
| shYBX1-1 | 5'- GAGAACCCTAAACCACAAGAT -3' |
| shYBX1-2 | 5'- GGTTCCCACCTTACTACAT -3'  -3' |
| shNOTCH1-1 | 5′- CAAAGACATGACCAGTGGCTA -3′ |
| shNOTCH1-2 | 5′- CGCTGCCTGGACAAGATCAAT -3′ |
| Probe sequences for RNA pulldown | |
| ACLY [C] | GAAAGAGCACCACCCTCTTCAGCCGCCACACCAAGGCCATTGTGTGGGGC-Biotin |
| ACLY [m5C] | GAAAGAGCACCACCCTCTTCAG[m5C]CGCCACACCAAGGCCATTGTGTGGGGC-Biotin |

**Supplemental Table-5** **Reagents and antibodies used in this study**

| **Antibody** | **Vendor** | **catalog number** |
| --- | --- | --- |
| DNMT2 | Abcam | ab308120 |
| ACLY | Santa Cruz  Proteintech | sc-365001  15421-1-AP |
| YBX1 | Proteintech | 20339-1-AP |
| α-Tubulin | Abclonal | A6830 |
| ACSS2 | Proteintech | 16087-1-AP |
| NOTCH1 | Proteintech | 20687-1-AP |
| HES1 | Abcam | ab71559 |
| HEY1 | Proteintech | 19929-1-AP |
| H4  H4ac  H3  H3ac  H3K9ac  H3K18ac  H3K27ac  Actinomycin D | CST  Abcam  CST  Abcam  CST  CST  CST  Sigma | #13919  ab177790  #4499  ab47915  #9649  #13998  #8173  A9415 |
| Sodium Acetate | Sigma | S5636 |
| TSA  C646 | MCE  MCE | HY-15144  HY-13823 |
| Lenvatinib  BMS-303141 | Selleck  MCE | #S1164  HY-16107 |

CST, Cell Signaling Technology;MCE, MedChemExpress
